# Ticagrelor induces paraoxonase-1 (PON1) and better protects hypercholesterolemic mice against atherosclerosis compared to clopidogrel

**DOI:** 10.1371/journal.pone.0218934

**Published:** 2019-06-26

**Authors:** Hasseri Halim, Decha Pinkaew, Preedakorn Chunhacha, Patuma Sinthujaroen, Perumal Thiagarajan, Ken Fujise

**Affiliations:** 1 Division of Cardiology, Department of Internal Medicine, University of Texas Medical Branch at Galveston, Galveston, Texas, United States of America; 2 Department of Pathology and Immunology, Baylor College of Medicine, Houston, Texas, United States of America; 3 Department of Biochemistry and Molecular Biology, University of Texas Medical Branch at Galveston, Galveston, Texas, United States of America; 4 The Institute of Translational Sciences, University of Texas Medical Branch at Galveston, Galveston, Texas, United States of America; Max Delbruck Centrum fur Molekulare Medizin Berlin Buch, GERMANY

## Abstract

Ticagrelor (TIC), a P2Y purinoceptor 12 (P2Y_12_)-receptor antagonist, has been widely used to treat patients with acute coronary syndrome. Although animal studies suggest that TIC protects against atherosclerosis, it remains unknown whether it does so through its potent platelet inhibition or through other pathways. Here, we placed hypercholesterolemic Ldlr^-/-^Apobec1^-/-^ mice on a high-fat diet and treated them with either 25 mg/kg/day of clopidogrel (CLO) or 180 mg/kg/day of TIC for 16 weeks and evaluated the extent of atherosclerosis. Both treatments equally inhibited platelets as determined by ex vivo platelet aggregation assays. The extent of atherosclerosis, however, was significantly less in the TIC group than in the CLO group. Immunohistochemical staining and ELISA showed that TIC treatment was associated with less macrophage infiltration to the atherosclerotic intima and lower serum levels of CCL4, CXCL10, and TNFα, respectively, than CLO treatment. Treatment with TIC, but not CLO, was associated with higher serum activity and tissue level of paraoxonase-1 (PON1), an anti-atherosclerotic molecule, suggesting that TIC might exert greater anti-atherosclerotic activity, compared with CLO, through its unique ability to induce PON1. Although further studies are needed, TIC may prove to be a viable strategy in the prevention and treatment of chronic stable human atherosclerosis.

## Introduction

Ticagrelor (TIC), originally known as AZD6140 (CAS #: 274693-27-5), is a cyclopentyl-triazolopyrimidine and a potent anti-platelet agent capable of directly and reversibly inhibiting the platelet P2Y_12_-receptor [1]. Both clopidogrel (CLO) and prasugrel (PRA), on the other hand, are thienopyridine prodrugs that require hepatic metabolism to generate their active metabolites to irreversibly bind and inhibit P2Y_12_ receptor [2]. These three platelet P2Y_12_-receptor antagonists—TIC, CLO, and PRA—have been widely used in the treatment of patients with acute coronary syndrome (ACS). In ACS, the rupture or erosion of atherosclerotic plaques leads to the drastic activation and aggregation of platelets, resulting in thrombosis of the coronary arteries, resulting in ischemia and necrosis of the distal myocardium [3]. TIC and PRO have been compared with CLO for their efficacy and safety in treating ACS. In the PLATelet inhibition and patient Outcomes (PLATO) trial, Wallentin et al. compared TIC (180 mg loading dose, 90 mg twice daily thereafter) and CLO (300–600 mg loading dose, 75 mg daily thereafter) for the prevention of cardiovascular events in patients with ACS [4]. They found that TIC, as compared with CLO, significantly reduced the rate of cardiovascular (CV) death, myocardial infarction (MI), or stroke without an increase in the rate of major bleeding [4]. In the PLATO PLATELET substudy, Storey et al. found that TIC more strongly suppressed platelet reactivity compared with CLO [5]. Because platelet activation and aggregation play a pivotal role in the pathogenesis of ACS [6], the superiority of TIC over CLO is likely due to its stronger P2Y_12_ receptor inhibition. This premise was supported by results of the Therapeutic Outcomes by Optimizing Platelet Inhibition With Prasugrel (TRITON) trial, in which PRA, a thienopyridine platelet antagonist more potent than CLO, outperformed CLO in reducing the rate of CV death, MI, or stroke [7].

Although more aggressive platelet inhibition using TIC and PRA led to better clinical outcomes than CLO in patients with ACS, it remains unclear whether the same holds true for non-ACS patients with stable atherosclerosis in which platelets contribute less to the progression and outcomes of the disease. Hiatt et al. randomized patients with symptomatic and stable peripheral artery disease (PAD) to either TIC (90 mg twice daily) or CLO (75 mg once daily) without aspirin (ASA) and followed them for 30 months in the Examining Use of Ticagrelor in Peripheral Artery Disease (EUCLID) trial [8]. They found that the primary efficacy end point consisting of CV death, MI, or ischemic stroke was similar between the TIC and CLO groups [8]. The degree and extent of atherosclerosis, however, was not assessed in the trial [8]. On the other hand, in the Prevention of Cardiovascular Events in Patients with Prior Heart Attack Using Ticagrelor Compared to Placebo on a Background of Aspirin–Thrombolysis in Myocardial Infarction 54 (PEGASUS-TIMI 54) trial, Bonaca et al. randomly assigned patients with a MI 1–3 years earlier to standard-dose TIC (90 mg twice daily), low-dose TIC (60 mg twice daily), or placebo, maintained them on low-dose ASA, followed them for 33 months, and found that both standard- and low-dose TIC significantly reduced the risk of CV death, MI, or stroke as compared with placebo [9]. However, there was no significant difference in the clinical outcome between standard- and low-dose TIC therapy [9]. In summary, there are no data to support the notion that TIC leads to better clinical outcomes or less atherosclerosis than CLO in patients with stable atherosclerosis [8], nor are there any data to support the premise that greater P2Y_12_ receptor inhibition leads to better clinical outcomes or less atherosclerosis in patients with stable atherosclerosis [9].

In experimental animals, P2Y_12_ antagonists have consistently been shown to protect against the progression of atherosclerosis. Heim et al. placed ApoE^-/-^ hyperlipidemic mice on a high fat diet (HFD containing 1% cholesterol, 7% fat, and 1% cholate), intraperitoneally injected them with 1 mg/kg CLO daily for 24 weeks, harvested their thoracic aortae, calculated the percentage of Sudan-IV-positive area to the whole vessel surface area, and found that CLO significantly decreased atherosclerosis in the thoracic aortae [10]. Mao et al. assigned ApoE^-/-^ hyperlipidemic mice for 16 weeks to four treatment groups: HFD alone, HFD plus 25 mg/kg/day of TIC, HFD plus 50 mg/kg/day of TIC, and HFD plus 100 mg/kg/day of TIC. They examined cross-sections of the ascending aortae of these mice and found that TIC at 100 mg/kg/day dosing, but not at 25 or 50 mg/kg/day dosing, significantly decreased atherosclerosis [11]. More recently, Preusch et al. administered 250 mg/kg/day of TIC to female ApoE^-/-^ mice fed normal chow and found that TIC significantly increased fibrous cap thickness and decreased the relative area of necrotic core, although there was no statistically different change in the degree of atherosclerosis [12]. Although none of these studies used standard whole aorta en face atherosclerosis assays to quantify the extent of atherosclerosis, their data suggest that both CLO and TIC, which are P2Y_12_ antagonists, inhibit the progression of atherosclerosis without ASA.

While both TIC and COL inhibit the P2Y_12_ receptor, TIC is structurally different from CLO and possesses biological activities that are not seen in CLO [13]. TIC, but not CLO, inhibits cellular uptake of adenosine by binding and inhibiting the equilibrative nucleoside transporter 1 (ENT1) [14] and increases extracellular levels of adenosine in humans [15, 16]. In addition, Thomas et al. reported that, compared with CLO, TIC decreased the peak levels of the pro-inflammatory cytokine IL-8 and increased those of the anti-inflammatory cytokine IL-10 in healthy volunteers who were challenged by endotoxin [17]. These observations, when taken together, suggest that some of the positive cardiovascular effects of TIC originate from biological activities other than anti-platelet activities [18, 19].

In the Dual Anti-Platelet Therapy (DAPT) study, Mauri et al. showed that DAPT beyond 1 year after placement of a drug-eluting stent (DES), as compared with ASA therapy alone, improved the cardiovascular outcomes of the patients with DES [20]. The study has led to the prolonged use of P2Y_12_ receptor antagonists in clinical practice. Hence, if a certain P2Y_12_ receptor antagonist better protects against atherosclerosis than the others, it is preferable for that antagonist to be used in patients with DES and atherosclerosis.

In the current study, we hypothesized that TIC ameliorates atherosclerosis more extensively than does CLO because of its biological effects unrelated to platelet inhibition. There have been no clinical or animal studies that definitively tested this clinically relevant hypothesis. We tested the hypothesis by placing Ldlr^-/-^Apobec1^-/-^ hypercholesterolemic mice on a HFD and treating them with either 180 mg/kg/d TIC or 25 mg/kg/d CLO, which we found were the doses that equally blocked platelet aggregation. We found that TIC (180 mg/kg/d), despite having the same degree of platelet inhibition as CLO (25mg/kg/d), better protected the mice against atherosclerosis. TIC, when compared with CLO, decreased the levels of pro-inflammatory cytokines CCL4 and CXCL10. Mechanistically, the aortae of TIC-treated animals expressed more paraoxonase-1 (PON1), an anti-atherosclerotic molecule, in the atherosclerotic intima and exhibited higher PON1 activities in the sera, suggesting a potential role of PON1 in the superior protection by TIC against atherosclerosis.

## Materials and methods

### Platelet aggregation assay

A platelet aggregation assay was performed using a hematology analyzer and as originally described by Ryan et al. [21] with some modification. Mice were fed with a normal chow diet or a diet containing TIC (180 mg/kg) or CLO (25 mg/kg) for 5 days. After 5 days, blood was collected from the inferior vena cava and diluted with D-Phenylalanyl-prolyl-arginyl Chloromethyl Ketone in phosphate buffered saline (PBS) to a final concentration of 0.08 mM. The diluted samples were mixed with (a) 20 mM ethylenediaminetetraacetic acid (EDTA) alone (the baseline tube) or (b) 20 mM EDTA, 30 μM adenosine diphosphate (ADP), and 3 μM 5-hydroxytryptamine (serotonin, the agonist tube). The mixture then was incubated at 37°C with shaking at 800 rpm for 4 min, after which a fixative solution was added. The fixed samples were counted using a Hemavet 950FS hematology analyzer (Drew Scientific, Inc., Dallas, TX), and the percentage of aggregation was calculated as ([total single platelet count (baseline tube)]–[platelet count after agonist treatment (agonist tube)])/(total single platelet count) x 100.

### Light transmission aggregometry

Platelet rich plasma (PRP) was obtained from the supernatant of a heparinized blood sample collected from the mice described above after a low-force centrifugation. The concentration of platelets in the PRP was adjusted to 300,000 platelets/μL by the addition of its own platelet poor plasma. Platelet aggregation was evaluated using a dual-channel aggregometer (Model 707, Chrono-Log Co., Havertown, PA) by adding 25 μM or 100 μM of ADP to the concentration-adjusted PRP as described previously [22].

### Platelet reactivity index (PRI)

The status of phosphorylation of the platelet vasodilator stimulated phosphoprotein (VASP) was measured using the PLT VASP/P2Y_12_ kit (BioCytex, Marseille, France) according to the manufacturer’s instructions as previously described [23]. Phosphorylation of VASP closely correlates with inhibition of the P2Y_12_ receptor and platelet aggregation [24]. Mice were fed with normal chow diet or a diet containing TIC (180 mg/kg) or CLO (25 mg/kg) for 5 days. Blood was collected from the inferior vena cava and treated with 0.109 M trisodium citrate by diluting nine parts of blood with one part of citrate. The citrated blood was mixed with either PGE1 or PGE1 + ADP and incubated at room temperature for 15 min. The platelets in the mixture were then fixed, permeabilized, immunolabeled with mouse anti-phosphorylated VASP (P-VASP) monoclonal antibody, and incubated at room temperature for 10 min. Cells were then incubated with anti-mouse IgG-FITC (to visualize P-VASP) and anti-CD61-PE (platelet) before they were subjected to flow cytometry (LSRFortessa Cell Analyzer, BD Biosciences, San Jose, CA). Data were analyzed using FlowJo (version 10, Ashland, OR). The mean fluorescence intensity (MFI) of the FITC signals of CD61-positive cells (platelets) was determined for PGE1-treated (MFI_PGE1_) and PGE1 + ADP-treated (MFI_PGE1+ADP_) cells [24]. A platelet reactivity index (RPI) was calculated as (MFI_PGE1_ –MFI_PGE1+ADP_)/ MFI_PGE1_. A higher RPI indicates more reactive platelets. The data are represented as mean ± standard deviation (SD) of four independent experiments.

### Tail blood loss measurement

Bleeding volume measurement was used to determine overall platelet function as described by Liu et al. [25] with minor modification. The mice were fed with a normal chow diet or a diet containing TIC (180 mg/kg) or CLO (25 mg/kg) for 5 days. Each mouse was then weighed before anesthesia, and a distal 5 mm segment of the tail was transected with a scalpel blade. The tail was immediately placed in a beaker filled with PBS prewarmed at 37°C. Bleeding was observed for 20 min, and the time to hemostasis was recorded. At the end of 20 min, each mouse was weighed again. Tail blood loss was calculated by the reduction in body weight.

### Blood pressure measurement

Mouse blood pressure was measured using the CODA mouse tail-cuff blood pressure system (Kent Scientific, Torrington, CT) according to the manufacturer's instructions and as described previously [26]. Briefly, the mice were restrained in a holder and placed on a warming platform to keep the body temperature constant. After the mice were acclimated for 5 min, the tails were placed with the occlusion and volume pressure recording (VPR) cuffs, which were connected to the system controller. The pressure was measured for 20 cycles per mouse.

### Cholesterol and triglyceride levels

Plasma levels of triglycerides, total cholesterol, phospholipids, and non-esterified fatty acids of mice were measured at the Mouse Metabolic Phenotyping Center at the University of Cincinnati as described previously [27].

### Serum TIC concentration assay

Serum TIC concentration was determined using high performance liquid chromatography-based methods as described previously [28, 29] at AstraZeneca R&D (Mölndal, Sweden).

### Atherosclerosis assay: Animals and diets

Eight-week-old male Ldlr^-/-^Apobec1^-/-^ hypercholesterolemic mice (C57BL/6J background) [26, 27, 30, 31] were randomly assigned to three groups: Control (CTL), Clopidogrel (CLO), and Ticagrelor (TIC). For the CTL group, a HFD (1.25% cholesterol, 40% kcal fat; D12108Ci [32]; Research Diets Inc., New Brunswick, NJ) was administered for 12 weeks to induce atherosclerosis. For the treatment groups, CLO (Millipore-Sigma, St. Louis, MO) and TIC (Astra-Zeneca) were added to the HFD at concentrations of 187.5 mg and 1350 mg per kilogram (kg), respectively, yielding the 25 mg/kg/d and 180 mg/kg/d doses for the mice weighing 30 g when fed 4 g/d. All mice were fed 4 g of the appropriate HFD daily.

### Atherosclerosis assay

#### En face atherosclerosis assay

The en face analysis of atherosclerosis was performed as described previously [26]. Briefly, the entire aorta, which included the distal portion of the ascending aortae, aortic arches, and descending aortae down to the iliac bifurcations, were pinned on a flat surface and fixed with 10% (v/v) buffered formalin solution overnight. The next day, the fixed aortae were stained with freshly prepared Oil Red O solution for 1 hour, destained twice with 78% methanol, and mounted on glass slides. Images were obtained by scanning the slides using the ScanScope slide scanning system (Nikon, Melville, NY). The entire areas of the aortae and Oil Red O-positive atherosclerotic lesions were measured using Image J software (NIH, Bethesda, MD) and expressed as percent lesion area.

#### Cross-sectional atherosclerosis assay

The cross-sectional analysis of atherosclerosis was performed as described previously [26]. Briefly, the proximal portion of the ascending aorta was embedded in the optimal cutting temperature compound (Sakura-Fineteck, Torrance, CA), frozen, and cut to obtain 5 μm cryostat sections containing the aortic valve leaflets. The sections were stained with hematoxylin and eosin (H&E), Oil Red O, and other immunostains. The H&E slides were scanned using the Motic EasyScan Digital Slide Scanner (Richmond, British Columbia, Canada). Image J software was used to quantify both the atherosclerotic lesion area and the total aortic sinus cross-sectional area in the same section at the level of the aortic valves. The cross-sectional lesion area was calculated by dividing the lesion area by the total aortic sinus cross-sectional area, and the value is expressed as a percentage [33].

#### Oil red O staining of the aortic roots

The 5 μm cryostat sections were fixed in 40% formaldehyde for 1 min and washed well in tap water. The sections were then stained in working Oil Red O solution for 10 min and counterstained in hematoxylin solution for 1 min. The 0.5% Oil Red O stock solution was made by dissolving 2.5 g Oil Red O powder in 500 ml isopropyl alcohol, and working solution was made by diluting the stock solution with distilled water at 3:2 (v/v), thoroughly mixing it, and then filtering it. The stained sections were mounted with aqueous mounting medium.

#### Immunohistochemistry (IHC) of the aortae

IHC of mouse aortae was performed as described previously [26] using the following primary antibodies and using 3,3′-diaminobenzidine (DAB) as the chromogen:

- F4/80 (Abcam, Cambridge, MA; ab6640, 1:200 dilution)
- α-SMA (Abcam; ab5694, 1:100 dilution)
- S100A4 (Abcam; ab41532, 1:500 dilution)
- TGFβ1 (Abcam; ab92486, 1:1000 dilution)
- 4-HNE (Abcam; ab48506, Clone HNEJ-2, 1:500 dilution)
- P-IRE1 (Abcam; ab48187, 1:4000 dilution)
- BAX (Abcam; ab32503, Clone E63, 1:200 dilution)
- Cleaved lamin A (Cell Signaling, Danvers, MA; 2035, 1:200 dilution)
- P-JNK (ThermoFisher Scientific, Rockford, IL; 700031, Clone D12H7L17, 1:50 dilution)
- PON1 (Novus Biologicals, Littleton, CO; NBP2-19893, 1:100 dilution)
- NOS1(Abcam; ab1376, 1:2000 dilution)
- ARG1 (Abcam; ab60176, 1:2000 dilution)

All immunostained sections were digitally imaged using the EasyScan Digital Slide Scanner (Motic, San Francisco, CA). Expression indices were calculated by dividing the DAB-positive area by the region of interest (ROI), and results are expressed as A.U. The cleavage of lamin is a well-characterized event in apoptosis [34].

### Multiplex serum chemokine/cytokine assay

The mouse serum cytokines and chemokines were simultaneously measured using a MILLIPLEX MAP Mouse Cytokine/Chemokine Magnetic Bead Panel according to manufacturer’s instructions (Catalog#: MCYTOMAG-70K, Millipore-Sigma, Burlington, MA). Briefly, the diluted serum samples, standards, and controls were mixed with assay buffer and premixed 32-plex Beads in a 96-well plate. The plate was then incubated at 4°C overnight with shaking. The well contents were removed, and the plate was washed twice with wash buffer. The mixture of detection antibodies was then added to each well and incubated for 1 hour at room temperature, followed by addition of streptavidin-phycoerythrin. After a 30 min incubation, the plate was washed twice and sheath fluid was added to each well prior to analysis using the Luminex 200 System (Luminex, Austin, TX).

### Next generation sequencing (NGS) of RNA from the mouse aortae

Aortae from the Ldlr^-/-^Apobec1^-/-^ mice treated with nothing (CTL, N = 5), CLO (N = 5), or TIC (N = 5) were excised from the body and cleaned to remove adjacent connective and adipose tissue. We then isolated RNA individually from each aorta as described previously using RNA purification columns (Qiagen, Germantown, MD) [35]. We examined the quality of the RNA using the Agilent 2100 Bioanalyzer (Santa Clara, CA) and found that all samples had RNA integrity numbers higher than 8. We prepared the library using the Illumina (San Diego, CA) TrueSeq mRNA sample preparation kit per the manufacturer’s instructions. RNA sequencing was performed using the Illumina HISeq 1500 machine at the University of Texas Medical Branch NGS Core facility. The sequencing run yielded about 15 million reads on average. We aligned all reads using Tophat with the mm10 build of the mouse genome reference and annotation from the University of Southern California downloaded from Illumina’s iGenome website. We then examined transcript assembly and differential expression using Cufflinks with Refseq mRNAs [36]. Finally, we analyzed the RNA-seq data using the cummerbund package in R [37].

### Ingenuity pathway analysis (IPA) of the aortae

The total RNAs from the aortae of mice on a HFD treated for 16 weeks with nothing (CTL, N = 5), CLO (N = 5), or TIC (N = 5) were subjected to RNA-sequencing using NGS. The Genbank IDs, Log_2_ fold changes (Log_2_FC), and expression P-values of the mapped genes from CLO vs. CTL (CLO/CTL) and TIC vs. CTL (TIC/CTL) groups were uploaded onto the Ingenuity Pathway Analysis (IPA, Qiagen) server. The IPA core analysis was performed focusing on the genes that were found to be differentially expressed at Log_2_FC at either > 0.6 or < –0.6 and *P* < 0.05. RT-qPCR analyses were used to confirm the pertinent observations from the RNA-Seq.

### IPA of the livers

To identify the cholesterol-regulating genes with expressions that were concordantly changed by CLO and TIC, we first performed a principal component analysis (PCA) on the data sets and found one of the CLO data sets (CLO27) to be an outlier (**S1 Fig**). We then uploaded the data (gene IDs, Log_2_FCs, and expression P-values) to the IPA server. We set cutoffs as follows: expression log ratio = 0.6 (52% increase or 48% decrease in expression levels) and adjusted expression P-value = 0.05. We found that 491 and 190, genes fit the criteria for the CLO/CTL and TIC/CTL data sets, respectively, which were submitted to the IPA core analyses. By running the IPA comparison analysis, we then identified eight genes that were significantly and concordantly perturbed in both the CLO/CTL and TIC/CTL groups. The data were visualized by the heatmaps generated by GraphPad Prism (GraphPad Software, Inc., La Jolla, CA).

### RT-qPCR

RT-qPCR was performed as described previously [38]. Briefly, the aortae and livers of CTL, CLO-, and TIC-treated Ldlr^-/-^Apobec1^-/-^ mice were harvested into Tri-Reagent (Molecular Research Center, Cincinnati, OH). RNA was isolated in accordance with the manufacturer’s instructions and treated with DNAse (ABI, Foster City, CA). RT-qPCR was performed in quadruplicate with exactly 50 ng of total RNA using the TaqMan RT-PCR kit (Applied Biosystems [ABI] at Life Technologies, Grant Island, NY) in the ABI Step One Plus Real-Time PCR system using the following primer and probe sets (Integrated DNA Technologies, Coralville, IA):

1. Mouse *PON1*—Forward: 5′-GGTCTTCCTATCAAACAAGATTAAATGC-3′, Reverse: 5′-AGTCTTCAGCACCCGTCTC -3′, Probe 5′-FAM-CCGTGAAGT/ZEN/AACGCCAGTAGAACTTCC -IAbkFQ-3′ where FAM = carboxyfluorescein, IAbkFQ = Iowa Black FQ, and ZEN = an internal quencher to enhance the quenching activity of the 3’ quencher Iowa Black FQ
2. Mouse *Early Growth Response 1* (*EGR1*)—Forward: 5′-AACAACCCTATGAGCACCTG-3′, Reverse: 5′-GAGTCGTTTGGCTGGGATAA-3′, Probe: 5′-FAM-AATGAGAAG/ZEN/GCGATGGTGGAGACG-IAbkFQ-3′ where FAM = carboxyfluorescein, IAbkFQ = Iowa Black FQ, and ZEN = an internal quencher to enhance the quenching activity of the 3’ quencher Iowa Black FQ
3. Mouse *18S rRNA*—Forward: 5′- GCCGCTAGAGGTGAAATTCT-3′, Reverse: 5′- TCGGAACTACGACGGTATCT-3′, Probe: 5′-JOEN- ACCAGAGCG/ZEN/AAAGCATTTGCCAAG-IAbkFQ-3′ where JOEN = 6-carboxy-4′,5′-dichloro-2′,7′- dimethoxyfluorescein, IAbkFQ = Iowa Black FQ, and ZEN = an internal quencher to enhance the quenching activity of the 3’ quencher Iowa Black FQ

### Serum PON1 activity assay

The activity of PON1 in mouse serum was quantified using the EnzChek Paraoxonase Assay Kit according to the manufacturer’s instructions (Catalog #: E33702, ThermoFisher Scientific) and as described previously [39]. The mouse serum samples were diluted by adding reaction buffer to each well of a 96-well plate. The freshly prepared 2X paraoxonase substrate working solution was then added to each well and thoroughly mixed. The plate was immediately transferred to a microplate reader set to 37°C, and the fluorescence was continuously read for 30 min using excitation at 360 nm and emission at 450 nm. The unit of paraoxonase activity was calculated from the amount of the fluorescent product as follows: 1 unit (U) of paraoxonase generates 1 nmol of fluorescent product per minute at 37°C.

### Western blot analyses using ProteinSimple WES

The WES is an automated capillary electrophoresis-based size fractionation and immunodetection system capable of providing high reproducibility, resolution, and sensitivity at a small sample volume and concentration (ProteinSimple, San Jose, CA, USA) [40–43]. Samples consisting of exactly 1 μg of total protein diluted in 5 μL of reaction mixture were loaded into the wells of the WES microplate. Blocking reagent, antibodies, chemiluminescent substrate, and wash buffer were also dispensed into designated wells on the microplate. The run was completed according to the manufacturer’s standard protocol. Anti-EGR1 (Abcam; ab133695, Clone EPR5014(2), 1:50 dilution) and anti-β-actin (Novus Biologicals; NB600-501SS, Clone AC-15, 1:25 dilution) antibodies were used to detect the respective proteins. Quantification of the signal was performed using Compass software (ProteinSimple), in which peak heights of the fluorescence signals were used to derive the relative EGR1 concentration for a given sample. The relative β-actin concentration for the same sample was calculated in the same way. The EGR1 expression index was calculated by dividing the relative EGR1 concentration by the relative β-actin concentration and normalizing the number from the liver treated by vehicle (CTL) to one.

### Ethics statement

This study was carried out in accordance with the recommendations in the Guide for the Care and Use of Laboratory Animals of the National Institutes of Health. All experiments involving animals were approved by the Institutional Animal Care and Use Committees (IACUC) of the University of Texas Medical Branch at Galveston. The experimental animals were regularly monitored for the entire duration of the experiment and at least once daily to identify signs of distress. Such distress was promptly addressed according to the IACUC guidelines. Isoflurane inhalation (until effective) followed by exsanguination (by aspiration of the blood from the heart or inferior vena cava) were used as the method of euthanasia.

### Statistical analysis

The degree of the spread of data was expressed by the SD. Student’s *t*-test was used to compare the means of two groups. To compare the means of three groups, analysis of variance (ANOVA) was employed with Fisher’s pairwise comparison, unless otherwise specified. P < 0.05 was considered to be statistically significant. P < 0.10 was considered to have a trend toward statistical significance. The numbers of mice used in in vivo experiments were determined by (i) power analysis, assuming an α error rate of 0.05, β error rate of 0.20, and expected difference of 25% and using Minitab 17 (State College, PA) or (ii) our previous dataset and experience from similar experiments performed in the past.

### Data availability statement

The authors declare that the data supporting the findings of this study are available within the paper and its supplementary information files. The entire raw and processed data from the RNA-Seq experiments on the aorta and liver of the hypercholesterolemic mice treated by TIC, CLO, or CTL are available on the ArrayExpress server with the accession numbers of E-MTAB-8050 and E-MTAB-8049, respectively. Finally, other relevant data are available from the authors upon request.

## Results

### CLO and TIC equally inhibit platelets at doses of 25 mg/kg/day and 180 mg/kg/day, respectively

To begin to test the hypothesis that TIC protects against atherosclerosis better than CLO due to its unique non-platelet-related biological activity, we first determined the dose of CLO that would exert the same anti-platelet activity as 180 mg/kg/day of TIC in mice. Mao et al. showed that TIC at a high dose (100 mg/kg/day), but not at low-intermediate doses (25 or 50 mg/kg/day), significantly ameliorated atherosclerosis in ApoE^-/-^ mice placed on a HFD (1% cholesterol and 5% lard) for 16 weeks [11]. However, because the investigators did not include CLO as a control, we did not know whether the anti-atherosclerotic effect of TIC was conferred by its anti-platelet function or by its unique non-platelet-related biological activity. In the current study that compared the anti-atherosclerotic activity of TIC, no drug (CTL), and CLO, we used 180 mg/kg/day of TIC to ensure that we could detect a difference in atherosclerosis, when present, between the TIC and CLO groups. C57BL/6J mice fed with normal chow were treated with no drug, 0, 25, 50, or 100 mg/kg/day of CLO, or 180 mg/kg/day of TIC for 5 days. After 5 days we obtained blood from the inferior vena cava to minimize platelet activation and subjected the samples to light transmission aggregometry. Platelets in CTL mice were aggregated by 25 μM adenosine diphosphate (ADP) (N = 6). Platelets from mice treated with any of the doses of CLO did not aggregate at either 25 or 100 μM ADP (N = 6), whereas one of the six samples from TIC-treated mice (N = 6) aggregated at 100 μM ADP. This result suggested that 25 mg/kg/day of CLO had at least equivalent anti-platelet activity as 180 mg/kg/day of TIC in mice (**S1A Fig**).

**Fig 1:**
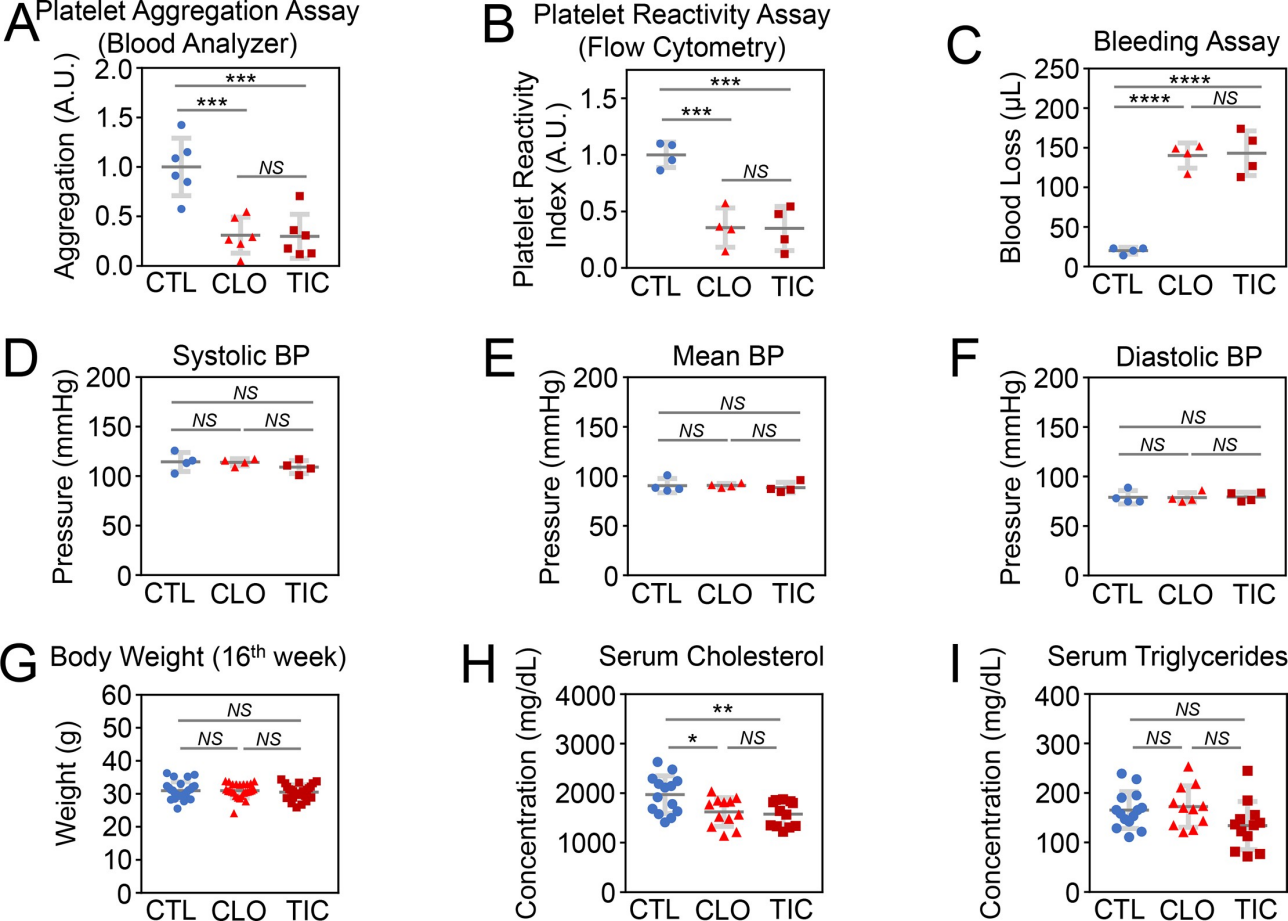
Clopidogrel and ticagrelor equally inhibit platelet aggregation at 25 mg/kg/day and 180 mg/kg/day, respectively. Abbreviations: CTL, control; CLO, clopidogrel; TIC, ticagrelor; A.U., arbitrary units; RPI, platelet reactivity index; BP, blood pressure; N = 4–6 per group for (A–F), 26 for (G), and 11–14 for (H–I); Error bars, means ± SD, statistical analyses performed using ANOVA with Fisher’s multiple comparison; NS, not statistically significant; *, P < 0.05; **, P < 0.01; ***, P < 0.005; ****, P < 0.001 *(See also S1 Fig)*. (A) Platelet aggregation assay using a hematology blood analyzer. (B) Platelet reactivity index (RPI) using flow cytometry. (C) Bleeding assay using tail blood loss measurement. (D) Systolic blood pressure measured. (E) Mean blood pressure calculated. (F) Diastolic blood pressure measured. (G) Body weights at 16^th^ week of treatment with a high-fat diet (HFD). (H) Serum cholesterol measured at 16^th^ week of treatment with a HFD. (I) Serum triglyceride measured at the 16^th^ week of treatment with a HFD.

Next, we determined relative platelet aggregation by adding ADP and 5-hydroxytryptamine (serotonin) to D-phenylalanyl-L-prolyl-L-arginine chloromethyl ketone-treated whole blood from mice treated for 10 days with either nothing (CTL), 25 mg/kg/day of CLO, or 180 mg/kg/day of TIC. Samples were incubated for 10 min then fixed with formaldehyde, and the number of aggregated platelets from each group was determined using the HEMAVET Hematology analyzer (N = 6 per group). TIC- and CLO-treated mice exhibited lower platelet aggregation than did CTL mice (**Fig 1A**). There was no significant difference in platelet aggregation between mice treated with 25 mg/kg/day of CLO and those treated with 180 mg/kg/day of TIC (**Fig 1A**). We then determined the status of VASP phosphorylation in platelets from animals in the CTL, CLO, and TIC (N = 4 per group) groups, using flow cytometry. Phosphorylation of VASP closely correlates with inhibition of the P2Y_12_ receptor and platelet aggregation [24]. We found that 25 mg/kg/day of CLO and 180 mg/kg/day of TIC equally inhibited the phosphorylation of platelet VASP (**Fig 1B**). Finally, we performed a tail bleeding assay (N = 4 per group) and found that mice treated with 25 mg/kg/d of CLO or 180 mg/kg/d of TIC exhibited the same degree of bleeding (**Fig 1C**). These data suggest that 25 mg/kd/day of CLO and 180 mg/kg/day of TIC equally prevent platelets from aggregating.

### TIC decreases atherosclerosis more than CLO in HFD-treated hypercholesterolemic mice

To test whether TIC better protects against atherosclerosis than does CLO when both are given in the doses that equally inhibit platelet aggregation, we performed a standard atherosclerosis assay by administering no drug (CTL), 25 mg/kg/day of CLO, or 180 mg/kg/day of TIC for 16 weeks to Ldlr^-/-^ Apobec1^-/-^ mice that were placed on a HFD. At the time of sacrifice, there was no significant difference in blood pressure (**Fig 1D, 1E & 1F**) or body weight among the three groups (**Fig 1G**). Sera from these animals were evaluated for cholesterol and triglyceride levels. Intriguingly, both CLO and TIC significantly decreased serum cholesterol levels compared to the CTL group, and there was no significant difference between CLO and TIC groups (**Fig 1H**, CTL vs. CLO vs. TIC = 1971 ± 382 vs. 1622 ± 290 vs. 1580 ± 255 mg/dL; N = 14, 12, and 12; P = 0.0096 for CTL vs. TIC; P = 0.0226 for CTL vs. CLO; not signifincant for CLO vs. TIC). Serum triglyceride levels were identical among CTL, CLO, and TIC groups (**Fig 1I**).

**Fig 2:**
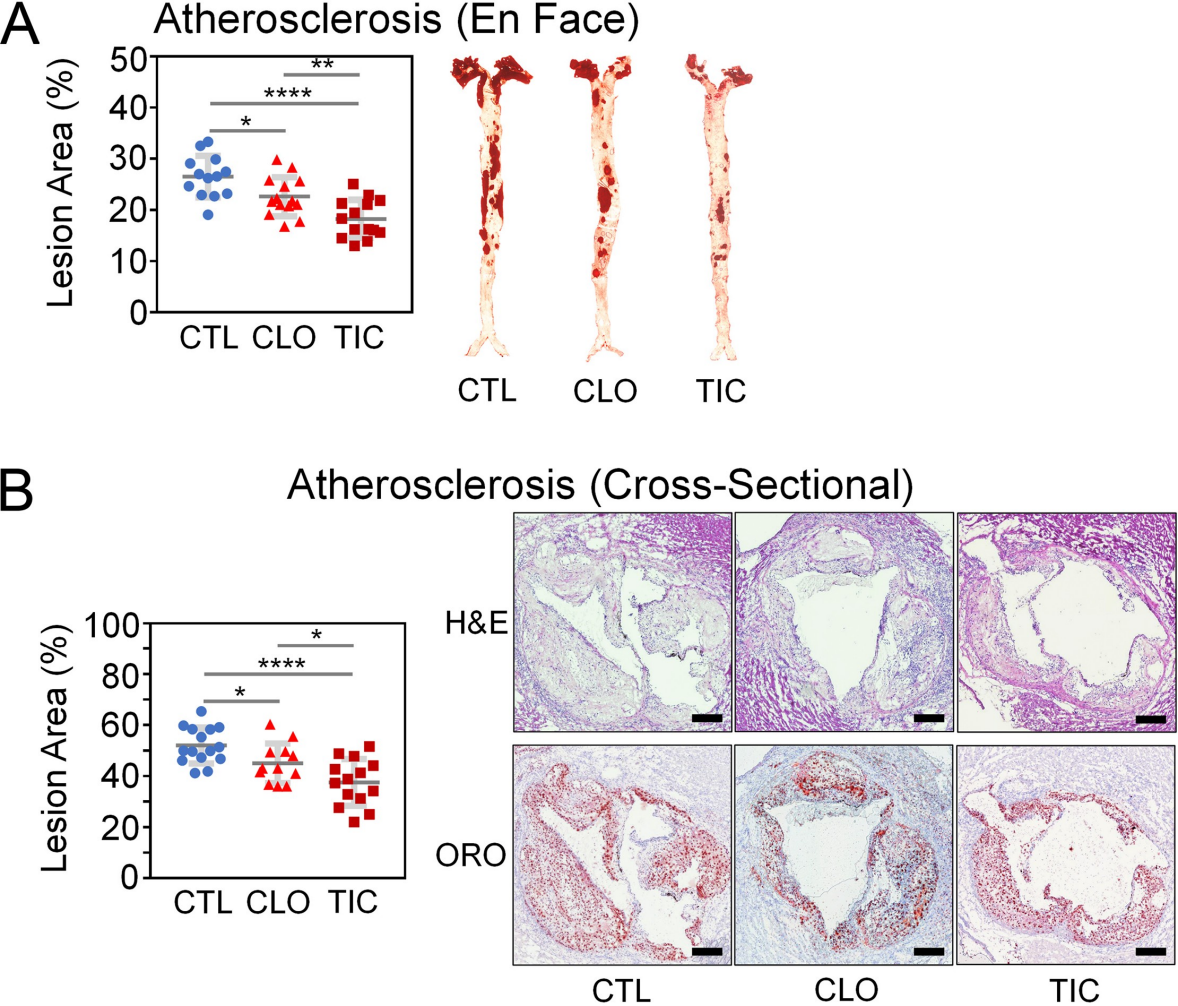
Ticagrelor exhibits superior protection against atherosclerosis compared to clopidogrel in the Ldlr^-/-^Apobec1^-/-^ hypercholesterolemic mice on a high-fat diet (HFD). Abbreviations: CTL, control; CLO, clopidogrel; TIC, ticagrelor; H&E, hematoxylin & eosin; ORO, oil red o; Size bars, 300 μm; N = 12–15 per group; Error bars, means ± SD, statistical analyses performed using ANOVA with Fisher’s multiple comparison; NS, not statistically significant; *, P < 0.05; **, P < 0.01; ****, P < 0.001 *(See also S2 Fig)*. Both en face (A) and cross-sectional (B) analyses show that TIC-treated animals were better protected against atherosclerosis than are CLO- or CTL-treated animals. CLO-treated animals exhibited less atherosclerosis than CTL-treated animals (B).

We then quantified the degree and extent of atherosclerosis using both the (a) en face assay of the entire aortae (**Fig 2A**) and (b) cross-sectional quantification of the atherosclerotic intima of the aorta roots at the level of aortic valves (**Fig 2B**)(N = 12–15 per group). Both CLO and TIC groups exhibited significantly less atherosclerosis than did the CTL group, and TIC treatment led to less atherosclerosis than did CLO treatment by both en face (**Fig 2A**, CTL vs. CLO vs. TIC = 26.48 ± 4.08 vs. 22.59 ± 3.78 vs. 18.26 ± 3.71; N = 13, 14, and 14; P = 0.013 for CTL vs. CLO; P < 0.001 for CTL vs. TIC; P = 0.005 for CLO vs. TIC, by ANOVA and Fisher pairwise comparison) and cross-sectional (**Fig 2B,** CTL vs. CLO vs. TIC = 52.03 ± 7.05 vs. 45.01 ± 7.74 vs. 37.54 ± 9.14; N = 15, 12, and 14; P = 0.03 for CTL vs. CLO; P < 0.001 for CTL vs. TIC; P = 0.023 for CLO vs. TIC, by ANOVA and Fisher pairwise comparison) assays. Serum TIC concentrations were 8.19 ± 4.23 μM (4,281.8 ± 2,225.4 ng/mL, biological replicates, N = 26)(**S2A Fig**), which were higher than what was reported in human pharmacokinetic studies [44–48].

### Both TIC and CLO treatments lead to decreased macrophage (MΦ) infiltration to atherosclerotic intima based on immunohistochemistry results

**Fig 3:**
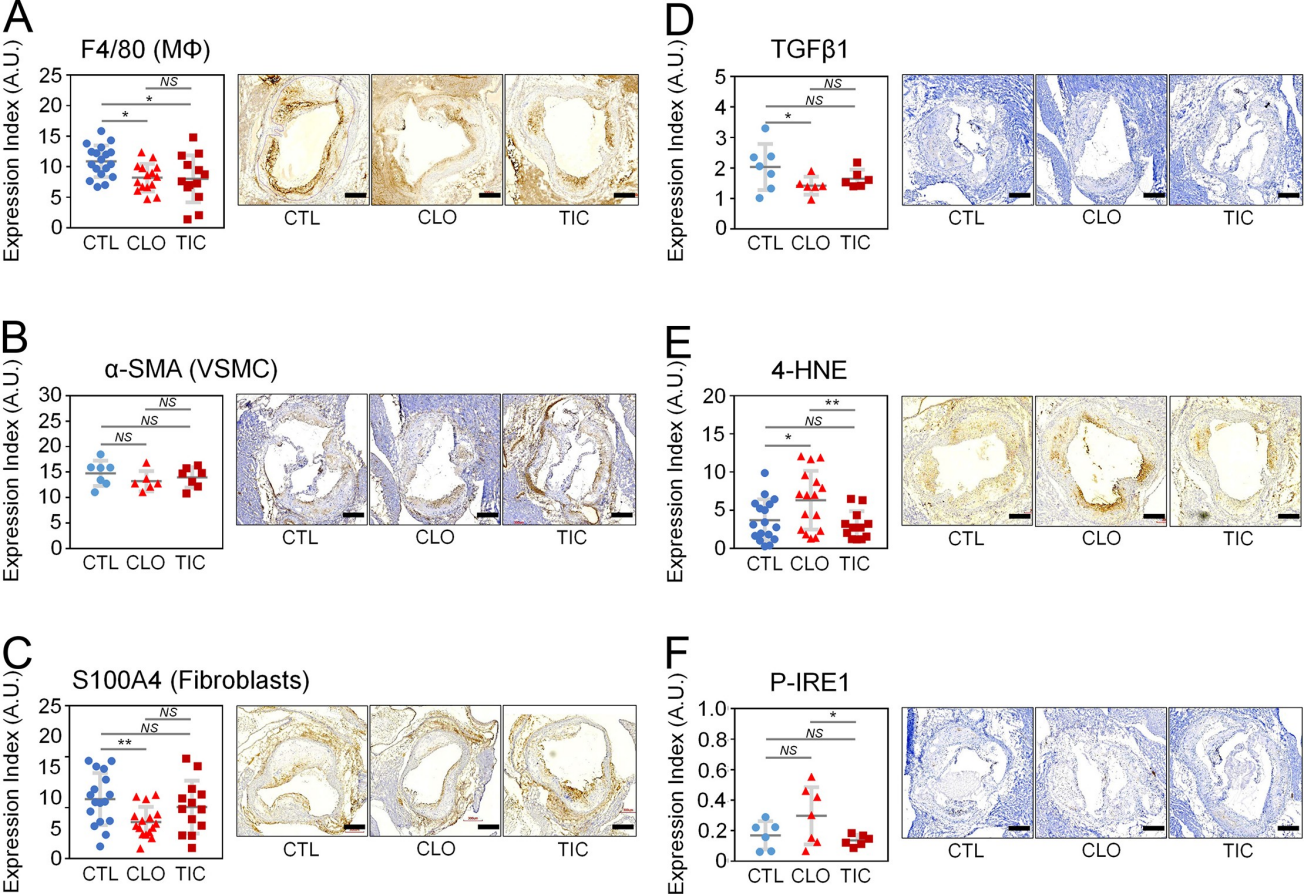
Immunohistochemical (IHC) analysis of the aortae of TIC-, CLO-, and CTL-treated animals. Abbreviations: CTL, control; CLO, clopidogrel; TIC, ticagrelor; A.U., arbitrary units; F4/80, F4/80 antigen also known as adhesion G protein-coupled receptor E1 (EMR1); MΦ, macrophages; α-SMA, alpha smooth muscle cell actin; VSMC, vascular smooth muscle cells; S100A4, S100 calcium-binding protein A4; FB, fibroblasts; TGFβ1, transforming growth factor β1; 4-HNE, 4-hydroxynonenal; P-IRE1, phosphorylated inositol-requiring enzyme 1; Size bars, 300 μm; N = 13–18, 6–7, 13–18, 6–7, 11–17, and 6–7 per group for (A), (B), (C), (D), (E), and (F), respectively; Error bars, means ± SD, statistical analyses performed using ANOVA with Fisher’s multiple comparison; NS, not statistically significant; *, P < 0.05; **, P < 0.01. *(See also S3 Fig)* (A) Decreased MΦ infiltration to the atherosclerotic intima of CLO- and TIC-treated compared to CTL-treated Ldlr^-/-^Apobec1^-/-^ mice placed on a HFD for 16 weeks as assessed by IHC staining using anti-F4/80 antibody. (B) No significant VSMCs migration to the intima of CTL-, CLO-, and TIC-treated mice. (C) Decreased fibroblasts in the intima of CLO-treated mice in comparison with CTL- and TIC-treated mice. (D) Decreased TGFβ1 in the intima of CLO-treated mice in comparison with CTL- and TIC-treated mice. (E) Increased 4-HNE lipid peroxidation marker in CLO-treated mouse aortae compared with CTL- and TIC-treated aortae. (F) Increased P-IRE1 ER stress marker in CLO-treated mouse aortae compared with TIC-treated aortae.

We sectioned the ascending aorta of the mice at the level of aortic valves or slightly above them and subjected them to IHC staining. Expression of F4/80, a MΦ antigen, was significantly lower in the atherosclerotic intima of CLO- and TIC-treated mice than in that of the CTL group (N = 13–18 per group, **Fig 3A)**, but there was no difference in expression between the CLO and TIC groups **(Fig 3A)**. Expression of α-smooth muscle cell actin (α-SMA), a vascular smooth muscle cell (VSMC)-specific antigen, did not differ among the CTL, CLO, and TIC groups (N = 6–7 per group, **Fig 3B**). Expression of S100 calcium binding protein A4 (S100A4), a fibroblast-specific protein, was significantly lower in the CLO than in the CTL or TIC groups, but there was no statistically significant difference in S100A4 expression between the CTL and TIC groups or between the CLO and TIC groups (N = 13–18 per group, **Fig 3C**). Expression of transforming growth factor 1 (TGFβ1), a pro-fibrosis factor [49], was significantly lower in the CLO than in the CTL or TIC groups, although there was no statistically significant difference in TGFβ1 expression between the CTL and TIC groups or between the CLO and TIC groups (N = 6–7 per group, **Fig 3D**). Expression of 4-hydroxy-2,3-E-nonenal (4-HNE), a marker of oxidative tissue damage, was significantly greater in the CLO group than in the CTL or TIC groups, but there was no statistically significant difference between the CTL and TIC groups (N = 11–17 per group, **Fig 3E**). Expression of phosphorylated inositol-requiring enzyme 1 (P-IRE1), a marker of endoplasmic reticulum stress, was significantly greater in the CLO group than the TIC group (P = 0.038); it also showed a trend toward greater expression in the CLO group than in the CTL group (P = 0.090), although there was no significant difference between the CTL and TIC groups (N = 7 for CLO; N = 6 for TIC and CTL, **Fig 3F**).

There was no difference among CTL, CLO, and TIC groups in the expression of the apoptosis markers Bax (**S3A Fig**), cleaved lamin A (**S3B Fig**), or phosphorylated c-Jun N-terminal kinase (P-JNK) [50] (**S3C Fig**), suggesting that CLO and TIC did not affect apoptosis in atherosclerotic intima. There was no difference among the CTL, CLO, and TIC groups in the expression of nitric oxide synthase 1 (NOS1), a M1 MΦ marker (N = 7 per group, **S3D Fig**), or arginase 1 (ARG1), a M2 MΦ marker (N = 7 per group, **S3E Fig**), suggesting that CLO and TIC did not affect the status of MΦ polarization in the atherosclerotic intima.

### TIC treatment, compared with CLO treatment, is associated with lower serum levels of proinflammatory chemokine (C-C motif) ligand 4 (CCL4), C-X-C motif chemokine 10 (CXCL10), and tumor necrosis factor alpha (TNFα) as shown by the multiplex cyto/chemokine assay

**Fig 4:**
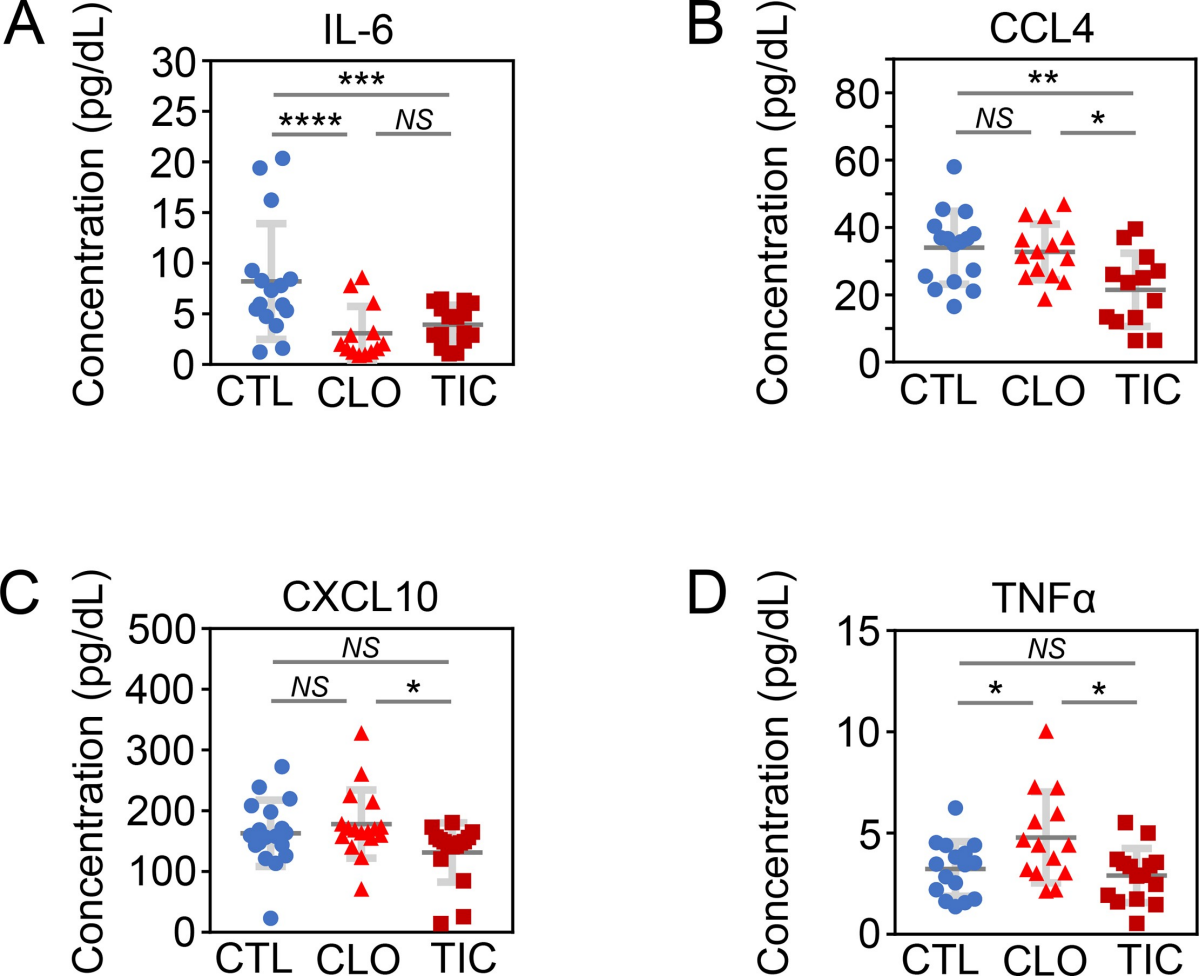
Serum chemo/cytokine profiles of CTL-, CLO-, and TIC-treated Ldlr^-/-^Apobec1^-/-^ mice placed on a HFD. Abbreviations: CTL, control; CLO, clopidogrel; TIC, ticagrelor; IL-6, interleukin 6; CCL4, C-C motif chemokine ligand 4 (also known as macrophage inflammatory protein 1β or MIP-1β); CXCL10, C-X-C motif chemokine ligand 10; TNFα, tumor necrosis factor alpha; N = 9–16, 13–16, 9–17, 15–16 per group, for (A), (B), (C), and (D), respectively; Error bars, means ± SD, statistical analyses performed using ANOVA with Fisher’s multiple comparison; NS, not statistically significant; *, P < 0.05; **, P < 0.01; ***, P < 0.005; ****, P < 0.001 *(See also S4 Fig).* (A) Decreased serum IL-6 levels in CLO- and TIC-treated mice compared with CTL-treated mice. (B) Decreased serum CCL4 levels in TIC-treated mice compared with CTL- and CLO-treated mice. (C) Increased serum CXCL10 levels in CLO-treated mice compared with TIC-treated mice. (D) Increased serum TNFα levels in CLO-treated mice compared with CTL- and TIC-treated mice.

To test whether the P2Y_12_ antagonists CLO and TIC affect the serum levels of cyto/chemokines, we subjected the sera from CTL-, CLO-, and TIC-treated mice to multiplex cyto/chemokine assays. Compared to CTL, both CLO and TIC significantly decreased levels of interleukin-6 (IL-6), a proinflammatory cytokine associated with atherosclerosis [51, 52]. However, no difference was detected in the cytokine levels between the CLO and TIC groups (**Fig 4A**). On the other hand, serum concentrations of the pro-inflammatory chemo/cytokines CCL4 [53], CXCL10 [54, 55, 56], and TNFα [57–59] were significantly lower in TIC-treated animals than in CLO-treated animals (**Fig 4B, 4C & 4D**). Levels of other anti-inflammatory (**S4, S4B, S4C, S4D, S4E** and **S4F Fig**) and pro-inflammatory (**S4G, S4H, S4I, S4J, S4K, S4L, S4M, S4N, S4O** and **S4P Fig**) cyto/chemokines did not differ among the CTL, CLO, and TIC groups.

### TIC, but not CLO, increases the message and protein levels of PON1, an anti-atherosclerotic molecule, in the atherosclerotic aortae

**Fig 5:**
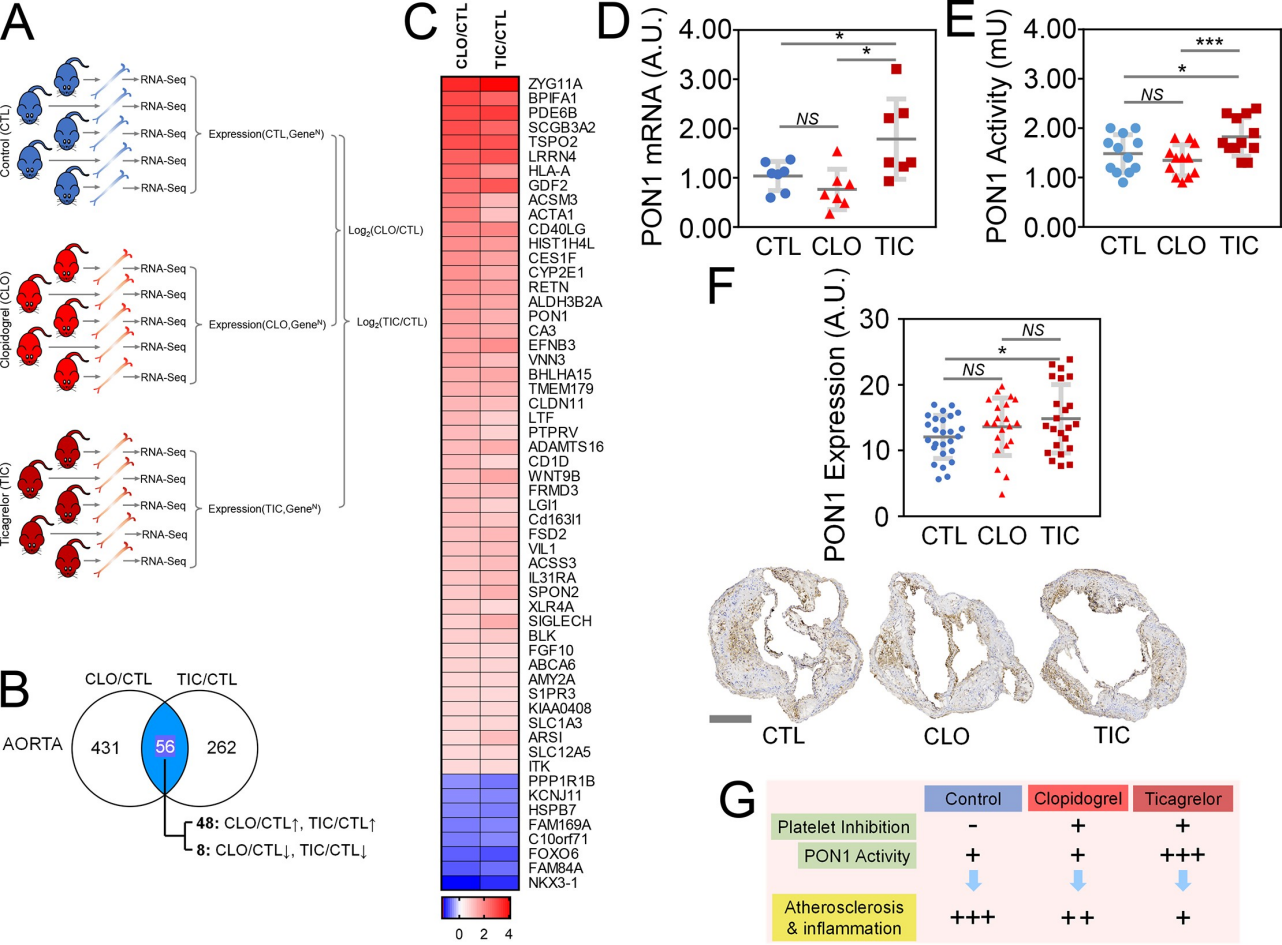
Ticagrelor treatment, but not clopidogrel treatment, induces the praroxonase-1 (PON1) message, increases tissue and serum PON1 protein, and mitigates atherosclerosis. Abbreviations: CTL, control; CLO, clopidogrel; TIC, ticagrelor; RNA-Seq, RNA sequencing using next generation sequencing; PON1, paraoxonase 1; A.U., arbitrary unit; N = 5 per group; Error bars, means ± SD, statistical analyses performed using one-way ANOVA test with Fisher’s multiple comparisons; *NS*, not statistically significant; *, P < 0.05; **, P < 0.01 *(See also S5 Fig).* (A) Experimental protocol to explore the mechanism(s) by which TIC decreases atherosclerosis more than does CLO. (B) Comparison of differentially expressed genes of the CLO/CTL group with those of the TIC/CTL group. Fifty-six genes were concordantly and differentially expressed in both CLO/CTL and TIC/CTL groups, of which 48 genes were concordantly upregulated and 8 concordantly downregulated in both groups. (C) List of genes that were concordantly and differentially expressed in both CLO/CTL and TIC/CTL groups. These are the genes that could explain the reduction of atherosclerosis by both CLO and TIC. Among these genes, only PON1 has been associated with the amelioration of atherosclerosis. (D) RT-qPCR confirming induction of the PON1 message by TIC compared with CTL and CLO. (E) Increased PON1 activity in the sera of TIC-treated Ldlr^-/-^Apobec1^-/-^ mice placed on a HFD compared with mice treated with CLO or CTL. (F) Increased tissue expression of PON1 in the atherosclerotic intima of TIC-treated mice compared with CTL-treated mice. (G) At the dose that inhibits platelets equally, TIC decreases atherosclerosis and associated inflammation more robustly than does CLO through its ability to induce PON1.

To explore the mechanism by which the P2Y_12_ antagonists CLO and TIC decreased atherosclerosis, we subjected the total RNAs from the aortae of mice on a HFD treated for 16 weeks with either nothing (CTL, N = 5), CLO (N = 5), or TIC (N = 5) to RNA-sequencing using NGS (**Fig 5A**). We uploaded the Genbank IDs, Log_2_ fold changes (Log_2_FC), and expression P-values of the 20,873 mapped genes from CLO vs. CTL (CLO/CTL) and TIC vs. CTL (TIC/CTL) groups to the IPA server. We then performed the IPA core analysis focusing on the genes that were found to be differentially expressed at (i) Log_2_FC > 0.6 or < –0.6 and (ii) *P* < 0.05. We found that 487 and 318 molecules from the CLO/CTL and TIC/CTL groups, respectively, met the above criteria and that 56 genes were differentially expressed in both CLO/CTL and TIC/CTL groups (**Fig 5B**). These genes could explain why both CLO- and TIC-treated animals exhibited less atherosclerosis than did CTL animals. Among the 56 genes, 48 and 8 genes were statistically significantly and concordantly regulated between the CLO/CTL and TIC/CTL groups, positively and negatively, respectively (**Fig 5C**). We then used the IPA Gene View to review each of the 56 genes, paying specific attention to their potential roles in atherosclerosis. None of the 8 concordantly downregulated genes was known to facilitate atherosclerosis. Among the 48 concordantly upregulated genes, only PON1 has been shown to ameliorate atherosclerosis [60] (**Fig 5C**). CCL4, CXCL10, and TNFα were not among the 56 genes (**Fig 5C**).

RT-qPCR analyses confirmed that the PON1 message level was significantly higher in TIC mouse aortae than in those of CTL and CLO mice (**Fig 5D**). However, the PON1 message level was not significantly different between CLO and CTL mouse aortae, suggesting that RNA-Seq data (**Fig 5A–5C**) overestimated a difference in PON1 expression between CLO and CTL. Consistently, serum PON1 activities were higher in TIC mouse sera than in both CLO and CTL mouse sera (CTL vs. CLO vs. TIC = 1.48 ± 0.38, 1.83 ± 0.39, and 1.35 ± 0.31; N = 12; P < 0.05 for CTL vs. TIC and CLO vs. TIC by ANOVA with multiple comparison using the Benjamini and Hochberg methods; **Fig 5E**). No difference in PON1 serum activity was detected between CLO and CTL mice (**Fig 5E**). Finally, quantitative IHC staining of the aortae showed that PON1 protein expression was significantly higher in TIC mouse aortae than in CTL mouse aortae, although there was no difference in PON1 protein expression between the TIC and CLO groups or between the CTL and CLO groups (**Fig 5F**).

### Treatment by TIC or CLO leads to lower serum cholesterol levels than treatment by CTL and is associated with suppression of EGR1 expression

Finally, we subjected the total RNAs from the livers of mice treated with either nothing (CTL, N = 3), CLO (N = 3), or TIC (N = 3) to RNA sequencing to explore the mechanism by which the P2Y_12_ antagonists CLO and TIC decrease serum cholesterol levels (**S5A Fig**). We uploaded the Genbank IDs, Log_2_FCs, and expression P-values of the 20,873 mapped genes from CLO vs. CTL (CLO/CTL) and TIC vs. CTL (TIC/CTL) groups to the IPA server. We then performed the IPA core analysis focusing on the genes that were found to be differentially expressed at (i) Log_2_FC > 0.6 or < –0.6 and (ii) P < 0.05. We found that 641 and 813 molecules from the CLO/CTL and TIC/CTL groups, respectively, met the above criteria (**S5B Fig**). We then compared the 641 genes from the CLO/CTL group with the 813 genes from the TIC/CTL group and found that 19 and 20 genes were concordantly regulated between the CLO/CTL and TIC/CTL groups, positively and negatively, respectively (**S5B Fig**). After reviewing these 39 genes for their potential link to hepatic lipid metabolism by surveying the available literature (**S5C Fig**), we found just one gene that would explain the anti-hypercholesterolemic phenotype of the TIC and CLO groups. This gene was EGR1. EGR1 binds to the promoter of the HMG-CoA reductase gene and transcriptionally activates the gene in response to insulin [61]. Mice deficient in the Egr1 gene (Egr1^-/-^ mice) exhibit significantly lower serum cholesterol levels [61].

To confirm that EGR1 was significantly downregulated in the TIC and CLO groups when compared with the CTL group, we performed WES (**S5D Fig**). Results showed that ERG1 levels were in fact significantly downregulated in the livers of TIC and CLO mice compared with those of CTL mice (**S5E Fig**) (CTL vs. CLO vs. TIC = 1.00 ± 0.102 vs. 0.64 ± 0.12 vs. 0.65 ± 0.18, N = 4 each, P < 0.05 for CTL vs. CLO and CTL vs. TIC; NS for CLO vs. TIC, by ANOVA with Fisher’s multiple comparisons test). Although further investigation is needed, these data suggest that both CLO and TIC reduced serum cholesterol levels by decreasing the expression of EGR1 in the liver.

## Conclusion

**Fig 6:**
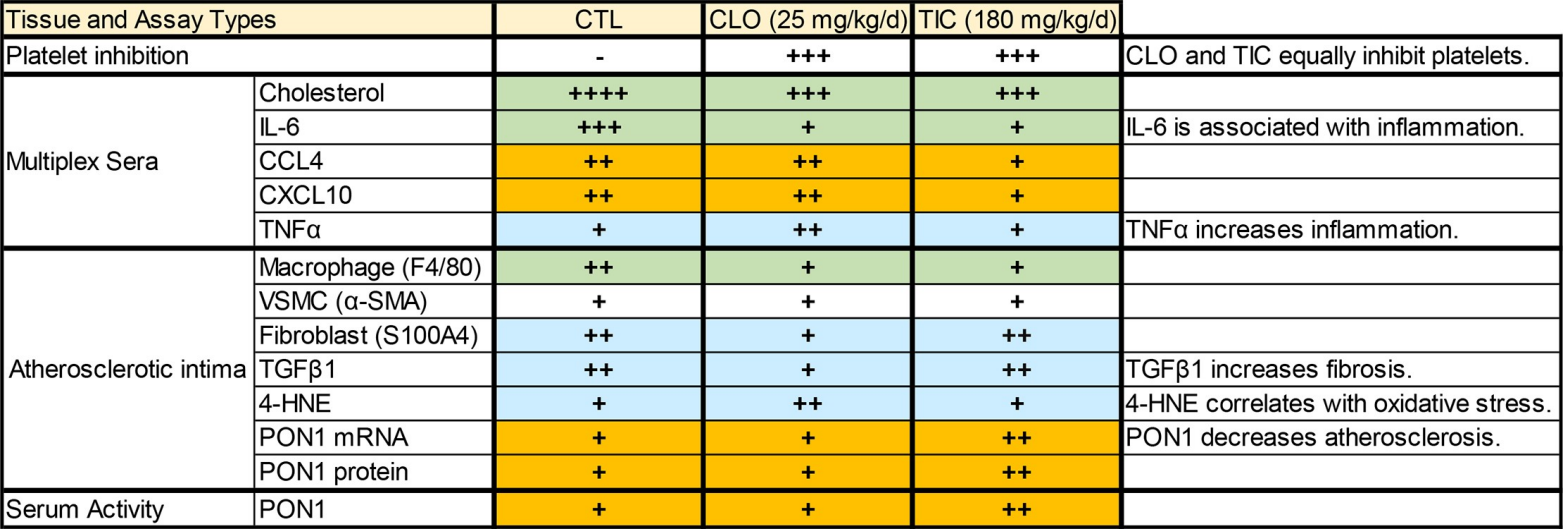
Biological phenotypes of CTL-, CLO-, and TIC-treated mice. Abbreviations: CTL, control; CLO, clopidogrel; TIC, ticagrelor; Orange, the results that could explain why TIC protects against atherosclerosis more than does CLO; Green, the results that could explain why TIC and CLO both protect against atherosclerosis, compared with CTL; Blue, the results that raise concerns on the possible negative vascular effects of CLO.

Our working model is depicted in **Fig 5G**, while the pertinent phenotypical differences among CTL, CLO, and TIC are summarized in **Fig 6**. TIC and CLO equally inhibited platelet aggregation at doses of 180 mg/kg/day and 25 mg/kg/day, respectively, in mice (**Fig 1A–1C** & **S1 Fig**). At these dosages, both CLO and TIC decreased atherosclerosis compared to no treatment (CTL) (**Fig 2A & 2B**) by blocking platelet activity and also by decreasing serum cholesterol levels (**Fig 1H**)—potentially through suppression of hepatic EGR1 expression (**S5A–S5E Fig**)—in Ldlr^-/-^Apobec1^-/-^ hypercholesterolemic mice. The reduction of atherosclerosis by CLO and TIC was associated with lower serum IL-6 levels (**Fig 4A**) and less MΦ infiltration to the atherosclerotic intima (**Fig 3A**). Strikingly, TIC decreased atherosclerosis more significantly than did CLO (**Fig 2A & 2B**), likely because TIC, unlike CLO, was able to induce the anti-atherosclerotic molecule PON1 [62–65] in the intima (**Fig 5D & 5F**) and to maintain higher serum PON1 activities in the mice (**Fig 5E**), which led to the lower levels of pro-inflammatory cytokines CCL4 and CXCL10 (**Fig 4B & 4C**).

## Discussion

The most significant finding of the current study is that although CLO and TIC equally inhibited platelet aggregation (**Fig 1A–1C**, **S1 Fig**), TIC protected hypercholesterolemic mice against atherosclerosis more robustly than did CLO (**Fig 2**) likely through the induction and increased serum activities of PON1 (**Fig 5A–5F**). PON1, a 354 amino acid glycoprotein associated with high-density lipoprotein (HDL), is considered to be a major factor in the anti-oxidative activity of HDL. PON1 is expressed in advanced atherosclerotic intima [62, 63], and PON1-deficient mice show an increased susceptibility to atherosclerosis [64, 65]. The finding that TIC treatment but not CLO treatment leads to greater expression of PON1 (**Fig 5D & 5F**) and higher serum activity of PON1 compared to the control (**Fig 5E**), when taken together with previous reports [62–65], suggests that the ability of TIC to induce PON1 in atherosclerotic arteries contributes to superior protection against atherosclerosis by TIC compared to CLO. However, the current data only support an association between TIC administration and PON1 induction. Further investigation is needed to definitively test the notion that TIC protects against atherosclerosis through PON1 induction. Although lower serum levels of CCL4, CXCL10, and TNFα in TIC-treated animals compared to CLO-treated animals (**Fig 4B, 4C & 4D**) could also explain the better protection by TIC than by CLO against atherosclerosis (**Fig 2A & 2B**), the lack of induction of these genes by either TIC or CLO (**Fig 5C**) makes them less likely candidates for the phenotype. Rather, the lower serum levels of those pro-inflammatory cyto/chemokines in TIC-treated animals, as compared with the CLO and/or CTL groups, likely reflect the lesser degree of inflammation and atherosclerosis in those animals.

We used Ldlr^-/-^Apobec1^-/-^ mice [27] for the mouse model of human atherosclerosis, as they represent the most scientifically rigorous model of human familial hypercholesterolemia with markedly elevated low density lipoprotein (LDL) levels [30, 31]. Although the total cholesterol level of ApoE^-/-^ mice is comparable to that of Ldlr^-/-^Apobec1^-/-^ mice (~560 mg/dL) and both strains develop robust atherosclerosis on normal chow [30], most of the cholesterol in ApoE^-/-^ mice resides in very low density lipoprotein and chylomicron (not in LDL). In contrast, most of the cholesterol of Ldlr^-/-^Apobec1^-/-^ mice resides in LDL [30]. ApoE deficiency could also result in defective phagocytosis of apoptotic cells and increased inflammation [66].

The finding that both CLO and TIC significantly decreased serum cholesterol levels in the HFD-fed Ldlr^-/-^Apobec1^-/-^ mice as compared with CTL (**Fig 1H**), potentially through suppression of EGR1 expression in the liver (**S5 Fig**) was unexpectedly discovered by NGS of the RNAs from the livers of CTL, CLO-treated, and TIC-treated mice and has not been previously reported. Further investigation is needed to elucidate exactly how CLO and TIC suppress EGR1 expression. In addition, more experiments are needed to demonstrate that CLO and TIC decrease serum cholesterol levels exclusively through their suppression of EGR1 expression in the liver. EGR1, also known as NGFI-A and KROX-24, is a 543 amino acid zinc finger transcription factor that is abundantly expressed in the liver and is a member of the early growth response family [61]. EGR1 is required for insulin-dependent induction of cholesterol genes such as HMG-CoA reductase, malic enzyme, and squalene epoxidase [61]. Egr1^-/-^ mice exhibit at least 50% lower serum cholesterol levels than do Egr1^+/+^ mice [61]. Although the cholesterol lowering effects of CLO and TIC may have contributed to their anti-atherosclerotic activity, the reduction by the P2Y_12_ receptor antagonists was mild at 18% and 20%, respectively, and may not be clinically relevant. In a similar atherosclerosis study, Heim et al. observed that parenteral treatment of HFD-fed ApoE^-/-^ mice with CLO (1 mg/kg/day, intraperitoneally) decreased serum cholesterol levels numerically, but the effect was not statistically significant [10]. Mao et al. measured serum cholesterol levels of HFD-fed ApoE^-/-^ mice treated with 0, 25, 50, and 100 mg/kg/day of TIC for 16 weeks and found no changes in the levels among these four groups of mice [11]. Preusch et al. assayed serum cholesterol levels of normal chow-fed ApoE^-/-^ mice treated with either nothing or 250 mg/kg/day of TIC and found no difference in serum cholesterol levels [12]. Other than the possibility that ApoE^-/-^ mice respond metabolically differently to the P2Y_12_ receptor antagonists than do our Ldlr^-/-^Apobec1^-/-^ mice, we do not know why other studies failed to show reduction by P2Y_12_ receptor antagonists of serum cholesterol levels in the hypercholesterolemic mice.

In the current study, multiplex cyto/chemokine analyses (**Fig 4**) as well as IHC analyses (**Fig 3**) raised some concerns about the impact of CLO on the vasculature, which was not seen in TIC-treated mice. First, the sera of CLO-treated mice had significantly higher TNFα than did sera from CTL or TIC-treated mice (**Fig 4D**). TNFα, one of the most potent pro-inflammatory cytokines, is present in human atherosclerotic plaques [57, 67]. Branen et al. placed ApoE^-/-^TNFα^+/+^ and ApoE^-/-^TNFα^-/-^ mice on a HFD for 10 weeks and found that ApoE^-/-^TNFα^-/-^ mice had a 50% reduction in atherosclerotic lesion area compared with ApoE^-/-^TNFα^+/+^ mice [59]. In addition, TNFα induces tissue factor (TF) in endothelial cells [68]. TF is not only abundantly expressed in human atherosclerotic plaques [69], but it also may facilitate atherosclerosis [70]. Reiner et al. showed that TIC, but not CLO, prevented TNFα from inducing TF in endothelial cells [71]. The inability of CLO to prevent TNFα from inducing TF [71], together with the higher serum TNFα levels seen in CLO treatment (**Fig 4D**), may have led to greater atherosclerosis in CLO-treated mice than in TIC-treated mice in the current study (**Fig 2**). However, this needs to be experimentally tested.

Second, our IHC analyses showed that CLO treatment was associated with lower expression of the fibrosis-promoting gene TGFβ1 and fibroblast-specific gene S100A4 compared to that seen in the CTL or TIC treatment groups (**Fig 3C & 3D**). Jia et al. induced cardiac fibrosis in mice by infusing angiotensin II in the presence or absence of CLO. They found that CLO treatment inhibited angiotensin II-induced cardiac fibrosis and TGFβ expression and concluded that platelet inhibition protected the heart against inflammation and fibrosis in response to angiotensin II [72, 73]. Our study, however, showed that TIC resulted in fibrosis and TGFβ expression in the atherosclerotic intima to a similar degree as that of CTL and less than that of CLO-treated animals (**Fig 3C & 3D**). This finding may suggest that CLO may have an off-target anti-fibrotic activity that is not present in TIC in the atherosclerotic intima. Further study is needed to investigate if and how CLO changes plaque composition and makes the atherosclerotic plaque more vulnerable to rupture by decreasing the ratio of fibrous tissue and lipids in the plaque [74].

Finally, CLO-treated mice exhibited significantly more 4-HNE expression in their atherosclerotic intima that did CTL and TIC-treated mice (**Fig 3E**). 4-HNE is a toxic aldehyde product of lipid peroxidation and a sensitive marker of oxidative damage and lipid peroxidation [75]. CLO, but not TIC, is a pro-dug that is metabolized first by cytochrome P450 (CYP) 1A2, 2C19, 2B6, and 3A4 and then by CYP2C19, 3A4, 2C9, and 2B6 to become an active metabolite containing a free thiol group that blocks the P2Y_12_ ADP receptor on the platelet surface by forming a disulfide bond with a cysteine residue of the receptor, hence preventing ADP from binding to the receptor [76]. Recent studies showed that these cytochrome P450 enzymes could metabolize CLO into other toxic, reactive intermediates that form off-target adducts and damage various cell types that express the CYPs [77, 78]. Further studies are needed to further evaluate the possibility of oxidative damage to vascular cells by CLO.

From the translational point of view, it is desirable for P2Y_12_ receptor antagonists to have anti-atherosclerotic effects, as they are almost always prescribed to patients with clinically significant atherosclerosis and its complications. The current experiments showed that TIC, when administered at a dose that inhibits platelet aggregation to the same degree as CLO, protects against atherosclerosis more robustly than CLO (**Fig 2A & 2B**) likely through its unique ability to induce PON1 (**Fig 5**). Further, TIC treatment did not lead to the potentially negative vascular phenotypes seen in CLO, such as higher TNFα serum levels (**Fig 4D**), lower fibroblast density (**Fig 3C**) and TGFβ1 expression (**Fig 3D**) that could make the plaque less stable, and higher levels of 4-HNE (**Fig 3E**). Despite the fact that TIC was not shown to be superior to CLO for the reduction of cardiovascular events in the EUCLID trial [8], the favorable vascular attributes of TIC found in the current study may justify additional clinical trials to test the anti-atherosclerotic effects of TIC compared with other P2Y_12_ antagonists.

## Supporting information

S1 Fig Traditional platelet aggregation assay using 200 μM adenosine diphosphate (ADP). Abbreviations: CTL, control; CLO, clopidogrel; TIC, ticagrelor; C57BL/6J mice were fed normal chow only or one with either 25 mg/kg/day of CLO or 180 mg/kg/day of TIC for 5 days. The blood from the mice was subjected to a standard platelet aggregation assay using an aggregometer and 25 or 100 μM ADP. All CTL samples aggregated with 25 μM ADP, whereas no CLO or TIC samples aggregated with 25 μM ADP (N = 6 per group). One out of six TIC samples and none of six CLO samples aggregated with 100 μM ADP *(See also Fig 1)*. *N* = 6 per group.
(PDF)

S2 Fig Mean ticagrelor (TIC) concentration of TIC-treated animals. Serum TIC concentrations were determined using high-performance liquid chromatography-based methods as described in the Methods section *(See also Fig 2). N* = 26 per group.
(PDF)

S3 Fig Additional immunohistochemical (IHC) analysis of the aortae of TIC-, CLO-, and CTL-treated animals. Abbreviations: CTL, control; CLO, clopidogrel; TIC, ticagrelor; A.U., arbitrary units; BAX, BCL2 associated X apoptosis regulator; P-JNK, phosphorylated mitogen-activated protein kinase 8; NOS1, nitric oxide synthase 1 and M1 inflammatory macrophage (MΦ) marker; ARG1, arginase 1 and M2 anti-inflammatory macrophage (MΦ) marker; Size bars, 300 μm; Error bars, means ± SD, statistical analyses performed using ANOVA with Fisher’s multiple comparison; NS, not statistically significant; *, *P* < 0.05, **, *P* < 0.01. *(See also Fig 3)*. No significant difference in BAX (A), cleaved lamin A (B), or P-JNK (C)—apoptosis markers—was detected in the atherosclerotic intima of CTL-, CLO-, and TIC- treated mouse aortae. No significant difference in NOS1 (M1 MΦ marker) (D) or ARG1 (M2 MΦ marker) was found in the atherosclerotic intima of CTL-, CLO-, and TIC-treated mouse aortae.
(PDF)

S4 Fig Additional serum chemo/cytokine profiles of CTL-, CLO-, and TIC-treated Ldlr^-/-^Apobec1^-/-^ mice placed on a HFD. Abbreviations: CTL, control; CLO, clopidogrel; TIC, ticagrelor; G-CSF, granulocyte colony stimulating factor (also known as CSF3, or colony stimulating factor 3); IL-1α, interleukin 1 alpha; IL-4, interleukin-4; IL-5, interleukin-5; IL-7, interleukin-7; IL-13, interleukin-13; IL-9, interleukin-9; CXCL1, C-X-C motif chemokine ligand 1; CXCL2, C-X-C motif chemokine ligand 2 (also known as macrophage inflammatory protein 2α (MIP-2α)); CXCL9, C-X-C motif chemokine ligand 9 (also known as monokine-induced by interferon-gamma (MIG)); CCL2, C-C motif chemokine ligand 2 (also known as monocyte chemoattractant protein-1 (MCP1)); CCL3, C-C motif chemokine ligand 3 (also known as macrophage inflammatory protein 1α (MIP-1α); CCL5, C-C motif chemokine ligand 5 (also known as regulated upon activation, normally T-expressed, and presumably secreted (RANTES)); CCL11, C-C motif chemokine ligand 11 (also known as eotaxin); CXCL5, C-X-C motif chemokine ligand 5 (also known as lipopolysaccharide-induced CXC chemokine (LIX)); VGEF, vascular endothelial growth factor A; *N* = 16–17, 16–17, 6–9, 13–18, 8, 14–17, 15–17, 16–17, 14–17, 16–18, 14–17, 14–16, 15–17, 15–18, 15–18, 13–17 per group, for (A)–(P), respectively; Error bars, means ± SD, statistical analyses performed using ANOVA with Fisher’s multiple comparison; NS, not statistically significant *(See also Fig 4).* No significant differences among CTL-, CLO-, and TIC-treated mice in the serum levels of G-CSF (A), IL-1α (B), IL-4 (C), IL-5 (D), IL-7 (E), IL-13 (F), IL-9 (G), CXCL1 (H), CXCL2 (I), CXCL9 (J), CCL2 (K), CCL3 (L), CCL5 (M), CCL11 (N), CXCL5 (O), and VGEF (P) were detected.
(PDF)

S5 Fig Clopidogrel and ticagrelor downregulate EGR1 expression in the mouse liver. Abbreviations: CTL, control; CLO, clopidogrel; TIC, ticagrelor; RNA-Seq, RNA sequencing using next generation sequencing; IB, immunoblot; A.U., arbitrary unit; Error bars, means ± SD, statistical analyses performed using one-way ANOVA test with Fisher’s multiple comparisons; *NS*, not statistically significant; *, *P* < 0.05; **, *P* < 0.01; *(See also Fig 5)*. (A) Experimental protocol to explore the mechanism(s) by which CLO and TIC decrease serum cholesterol levels (N = 3 per group). (B) Comparison of differentially expressed genes of the CLO/CTL group with those of the TIC/CTL group. Thirty-nine genes were concordantly and differentially expressed in both CLO/CTL and TIC/CTL groups, of which 19 genes were concordantly upregulated and 20 concordantly downregulated in both groups. (C) List of the genes that were concordantly and differentially expressed in both the CLO/CTL and TIC/CTL groups. These are the genes that could explain the reduction of serum cholesterol levels by both CLO and TIC. (D) WES-based Western blot analysis confirming the reduction of EGR1 by both CLO and TIC. EGR1 binds the promoter of the HMG-CoA- reductase gene. Downregulation of EGR1 leads to lower HMG-CoA-reductase gene expression and cholesterol synthesis in the liver. (E) Quantification of the EGR1 signals showing the significant reduction of EGR1 expression by both CLO and TIC in the liver.
(PDF)

S6 Fig Cropped and full-length blots. Abbreviations: CTL, control; CLO, clopidogrel; TIC, ticagrelor; (A) S5D Fig. (B) Full-length α-EGR1 blots, from which a cropped portion (red rectangle) was taken to generate the EGR1 portion of S5D Fig. (C) Full-length α-β-Actin blots, from which a cropped portion (red rectangle) was taken to generate the β-Actin portion of S5D Fig.
(PDF)
