# Supplementary figures and images for: Ticagrelor induces paraoxonase-1 (PON1) and better protects hypercholesterolemic mice against atherosclerosis compared to clopidogrel
Source: PLoS One. 2019 Jun 26;14(6):e0218934. doi: 10.1371/journal.pone.0218934 (PMC6594647; doi:10.1371/journal.pone.0218934)

A

100  $\mu$ M ADP  
 Aggregated  
 Not aggregated

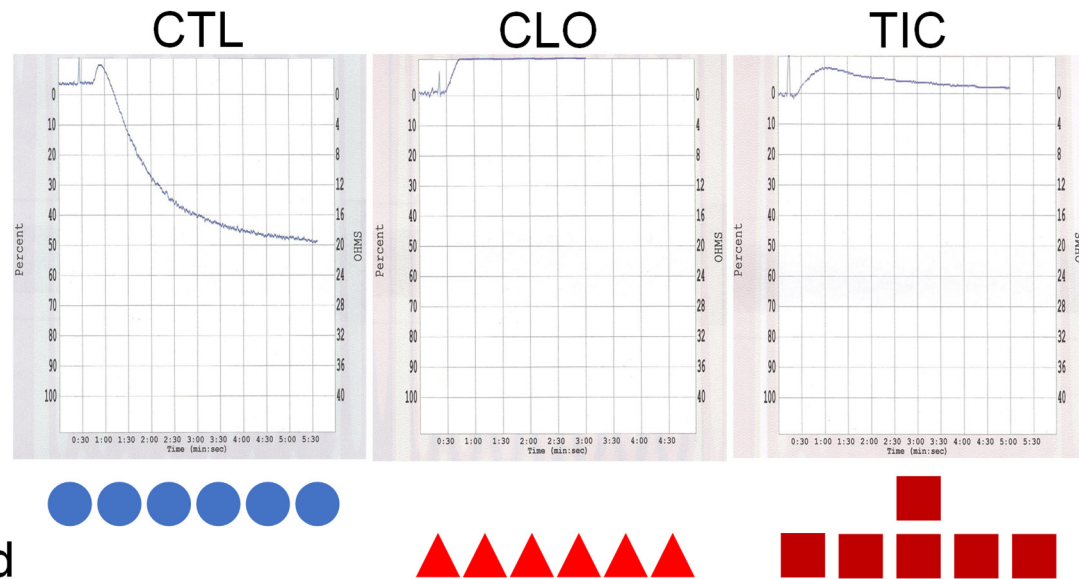

(S1 Fig)

Supplement: S1 Fig — Abbreviations: CTL, control; CLO, clopidogrel; TIC, ticagrelor; C57BL/6J mice were fed normal chow only or one with either 25 mg/kg/day of CLO or 180 mg/kg/day of TIC for 5 days. The blood from the mice was subjected to a standard platelet aggregation assay using an aggregometer and 25 or 100 μM ADP. All CTL samples aggregated with 25 μM ADP, whereas no CLO or TIC samples aggregated with 25 μM ADP (N = 6 per group). One out of six TIC samples and none of six CLO samples aggregated with 100 μM ADP (See also Fig 1). N = 6 per group. (PDF) [file pone.0218934.s001.pdf]

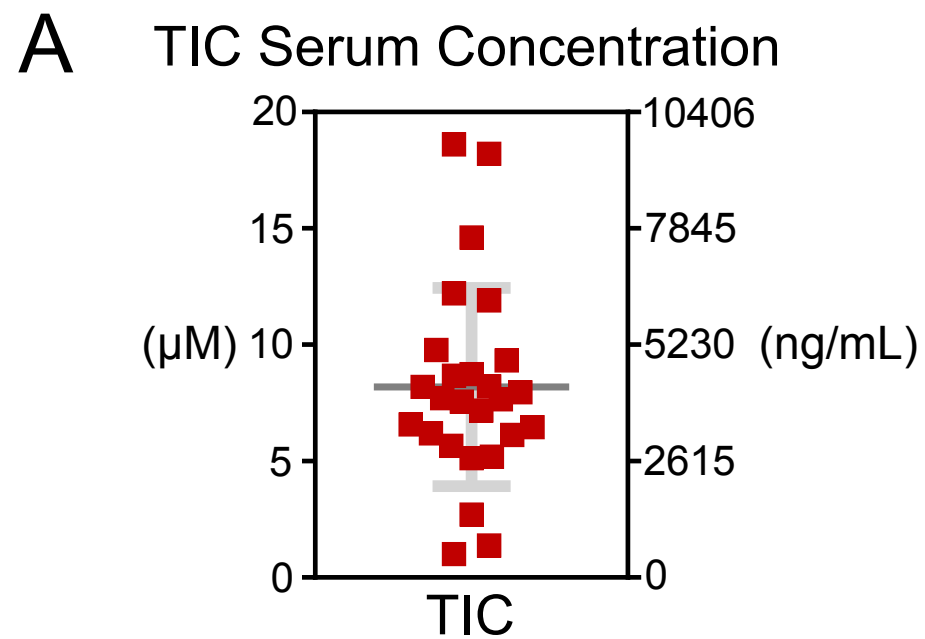

**(S2 Fig)**

Supplement: S2 Fig — Serum TIC concentrations were determined using high-performance liquid chromatography-based methods as described in the Methods section (See also Fig 2). N = 26 per group. (PDF) [file pone.0218934.s002.pdf]

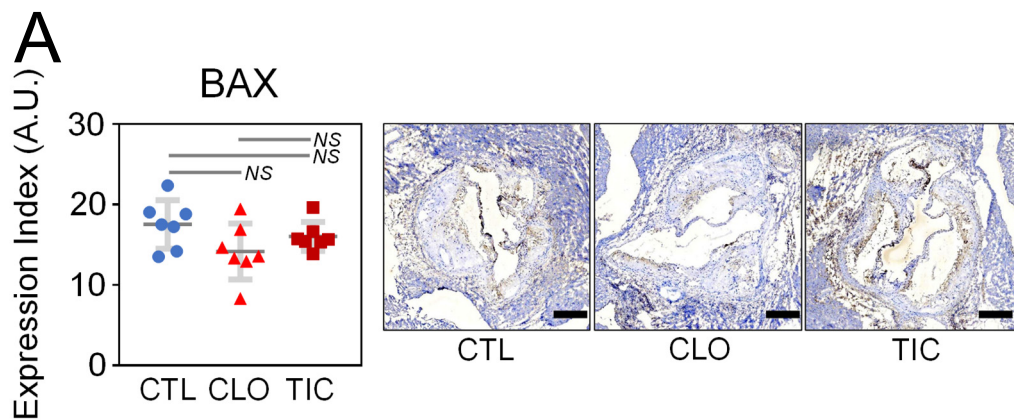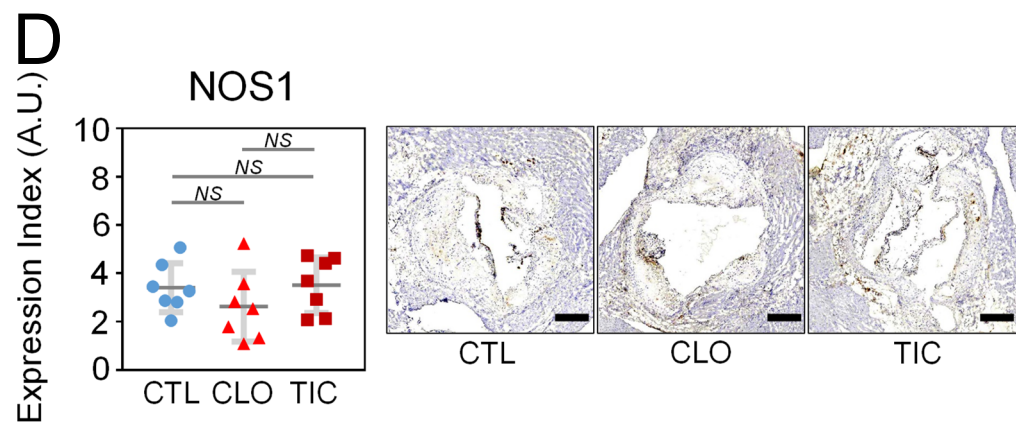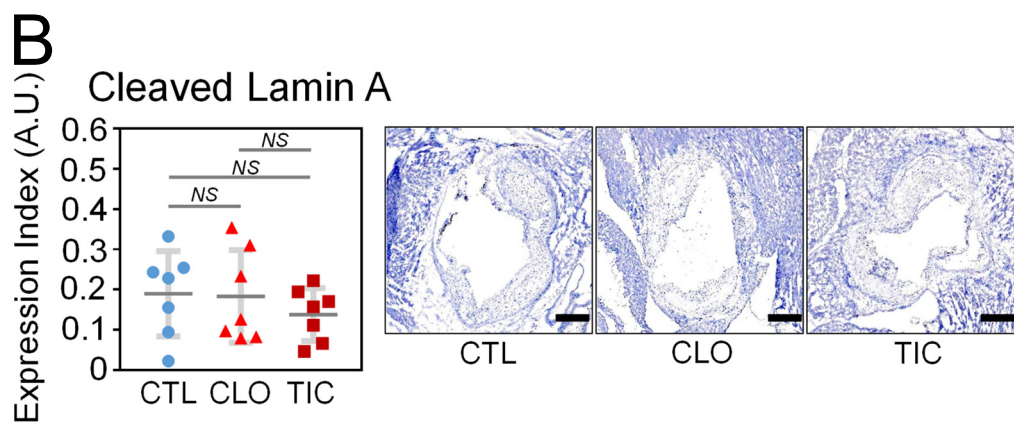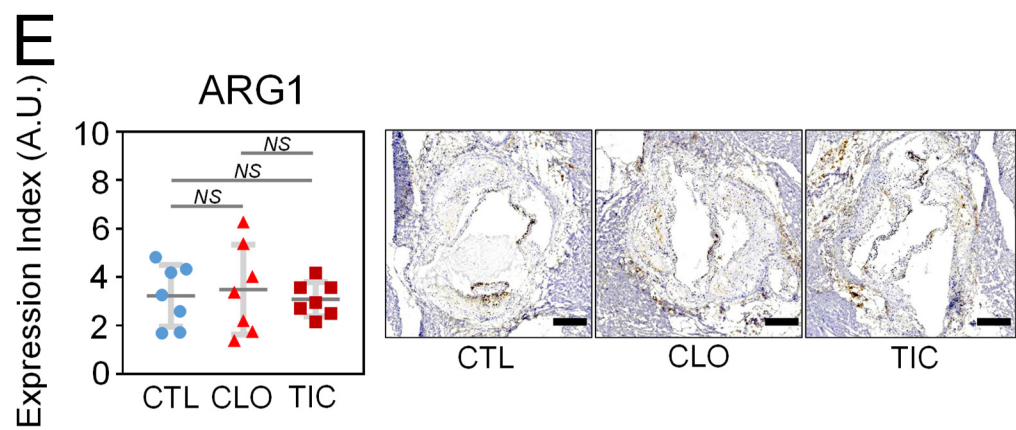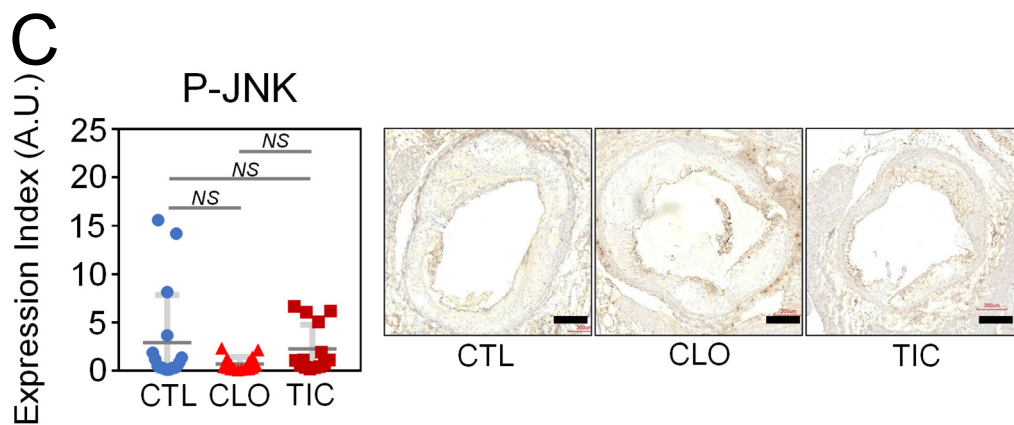

(S3 Fig)

Supplement: S3 Fig — Abbreviations: CTL, control; CLO, clopidogrel; TIC, ticagrelor; A.U., arbitrary units; BAX, BCL2 associated X apoptosis regulator; P-JNK, phosphorylated mitogen-activated protein kinase 8; NOS1, nitric oxide synthase 1 and M1 inflammatory macrophage (MΦ) marker; ARG1, arginase 1 and M2 anti-inflammatory macrophage (MΦ) marker; Size bars, 300 μm; Error bars, means ± SD, statistical analyses performed using ANOVA with Fisher’s multiple comparison; NS, not statistically significant; *, P < 0.05, **, P < 0.01. (See also Fig 3). No significant difference in BAX (A), cleaved lamin A (B), or P-JNK (C)—apoptosis markers—was detected in the atherosclerotic intima of CTL-, CLO-, and TIC- treated mouse aortae. No significant difference in NOS1 (M1 MΦ marker) (D) or ARG1 (M2 MΦ marker) was found in the atherosclerotic intima of CTL-, CLO-, and TIC-treated mouse aortae. (PDF) [file pone.0218934.s003.pdf]

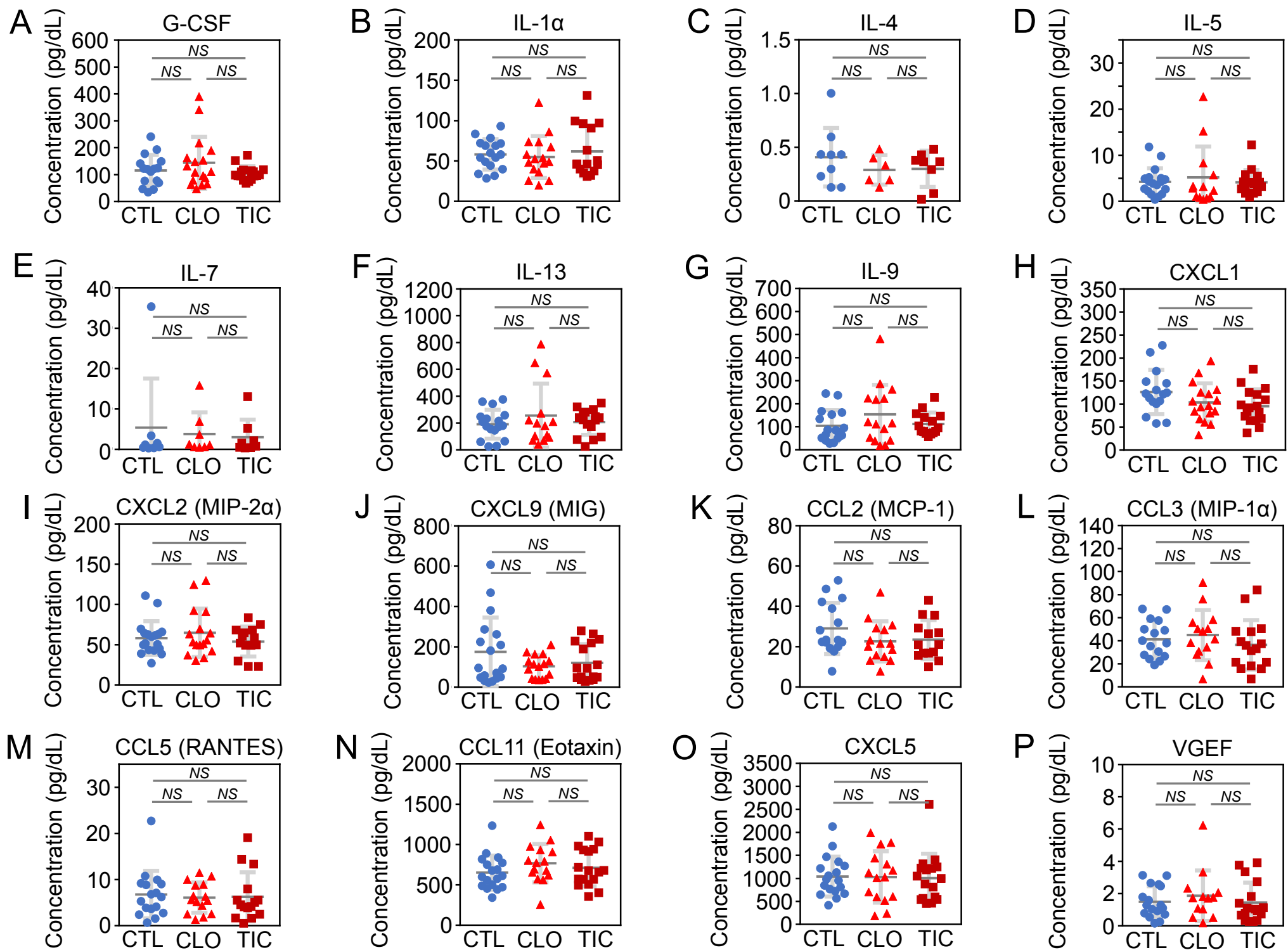

(S4 Fig)

Supplement: S4 Fig — Abbreviations: CTL, control; CLO, clopidogrel; TIC, ticagrelor; G-CSF, granulocyte colony stimulating factor (also known as CSF3, or colony stimulating factor 3); IL-1α, interleukin 1 alpha; IL-4, interleukin-4; IL-5, interleukin-5; IL-7, interleukin-7; IL-13, interleukin-13; IL-9, interleukin-9; CXCL1, C-X-C motif chemokine ligand 1; CXCL2, C-X-C motif chemokine ligand 2 (also known as macrophage inflammatory protein 2α (MIP-2α)); CXCL9, C-X-C motif chemokine ligand 9 (also known as monokine-induced by interferon-gamma (MIG)); CCL2, C-C motif chemokine ligand 2 (also known as monocyte chemoattractant protein-1 (MCP1)); CCL3, C-C motif chemokine ligand 3 (also known as macrophage inflammatory protein 1α (MIP-1α); CCL5, C-C motif chemokine ligand 5 (also known as regulated upon activation, normally T-expressed, and presumably secreted (RANTES)); CCL11, C-C motif chemokine ligand 11 (also known as eotaxin); CXCL5, C-X-C motif chemokine ligand 5 (also known as lipopolysaccharide-induced CXC chemokine (LIX)); VGEF, vascular endothelial growth factor A; N = 16–17, 16–17, 6–9, 13–18, 8, 14–17, 15–17, 16–17, 14–17, 16–18, 14–17, 14–16, 15–17, 15–18, 15–18, 13–17 per group, for (A)–(P), respectively; Error bars, means ± SD, statistical analyses performed using ANOVA with Fisher’s multiple comparison; NS, not statistically significant (See also Fig 4). No significant differences among CTL-, CLO-, and TIC-treated mice in the serum levels of G-CSF (A), IL-1α (B), IL-4 (C), IL-5 (D), IL-7 (E), IL-13 (F), IL-9 (G), CXCL1 (H), CXCL2 (I), CXCL9 (J), CCL2 (K), CCL3 (L), CCL5 (M), CCL11 (N), CXCL5 (O), and VGEF (P) were detected. (PDF) [file pone.0218934.s004.pdf]

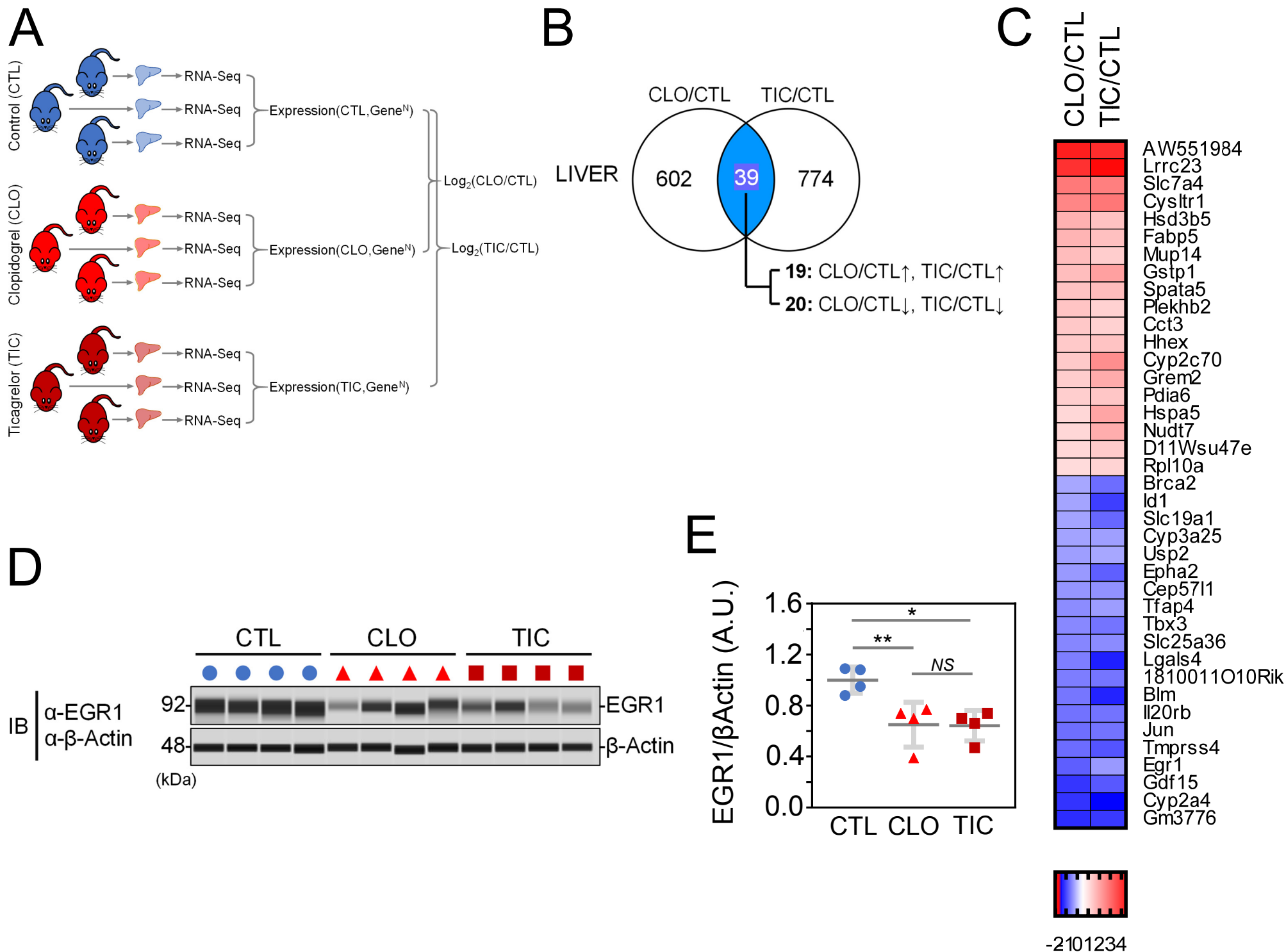

(S5 Fig)

Supplement: S5 Fig — Abbreviations: CTL, control; CLO, clopidogrel; TIC, ticagrelor; RNA-Seq, RNA sequencing using next generation sequencing; IB, immunoblot; A.U., arbitrary unit; Error bars, means ± SD, statistical analyses performed using one-way ANOVA test with Fisher’s multiple comparisons; NS, not statistically significant; *, P < 0.05; **, P < 0.01; (See also Fig 5). (A) Experimental protocol to explore the mechanism(s) by which CLO and TIC decrease serum cholesterol levels (N = 3 per group). (B) Comparison of differentially expressed genes of the CLO/CTL group with those of the TIC/CTL group. Thirty-nine genes were concordantly and differentially expressed in both CLO/CTL and TIC/CTL groups, of which 19 genes were concordantly upregulated and 20 concordantly downregulated in both groups. (C) List of the genes that were concordantly and differentially expressed in both the CLO/CTL and TIC/CTL groups. These are the genes that could explain the reduction of serum cholesterol levels by both CLO and TIC. (D) WES-based Western blot analysis confirming the reduction of EGR1 by both CLO and TIC. EGR1 binds the promoter of the HMG-CoA- reductase gene. Downregulation of EGR1 leads to lower HMG-CoA-reductase gene expression and cholesterol synthesis in the liver. (E) Quantification of the EGR1 signals showing the significant reduction of EGR1 expression by both CLO and TIC in the liver. (PDF) [file pone.0218934.s005.pdf]

**A**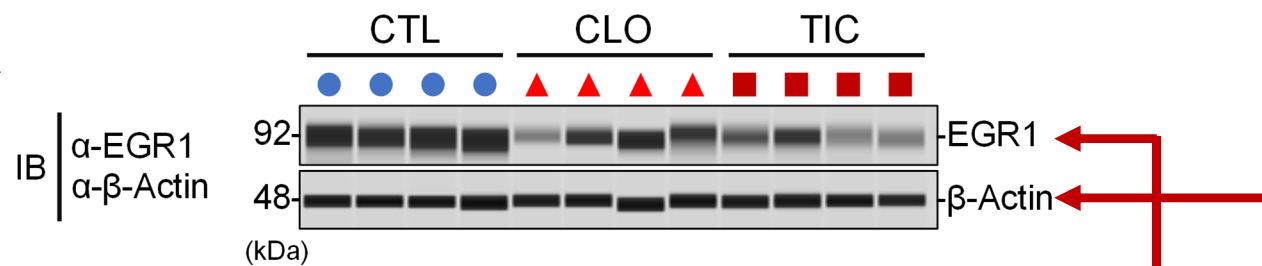**B**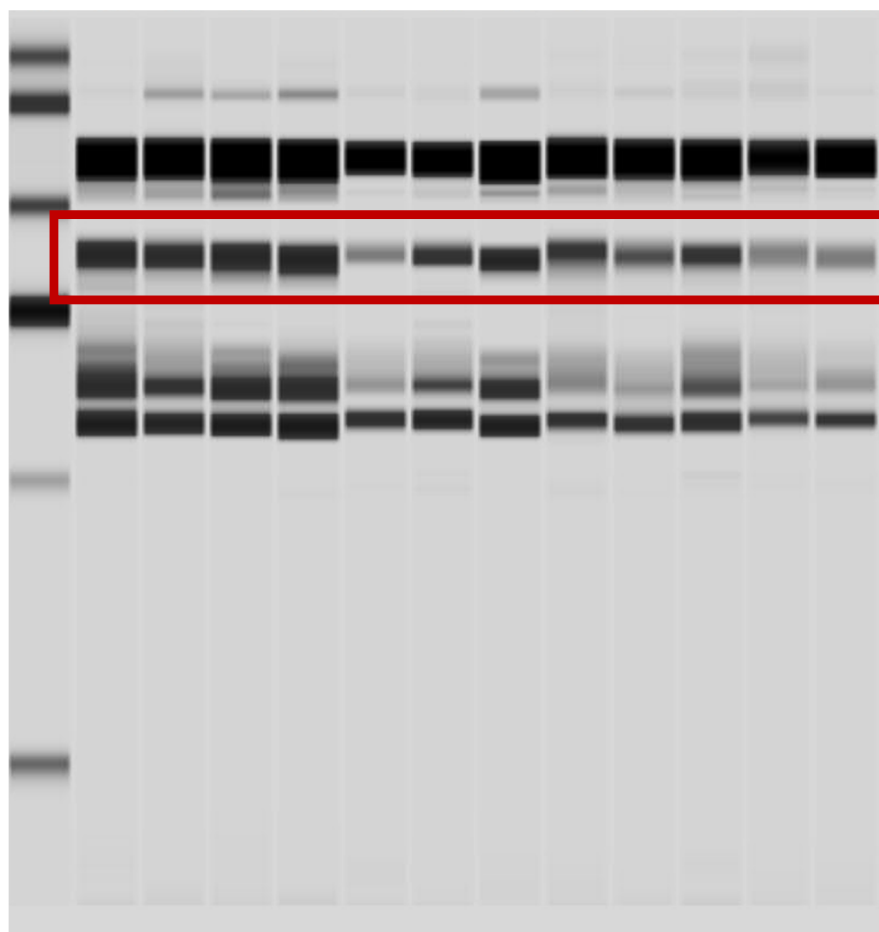**C**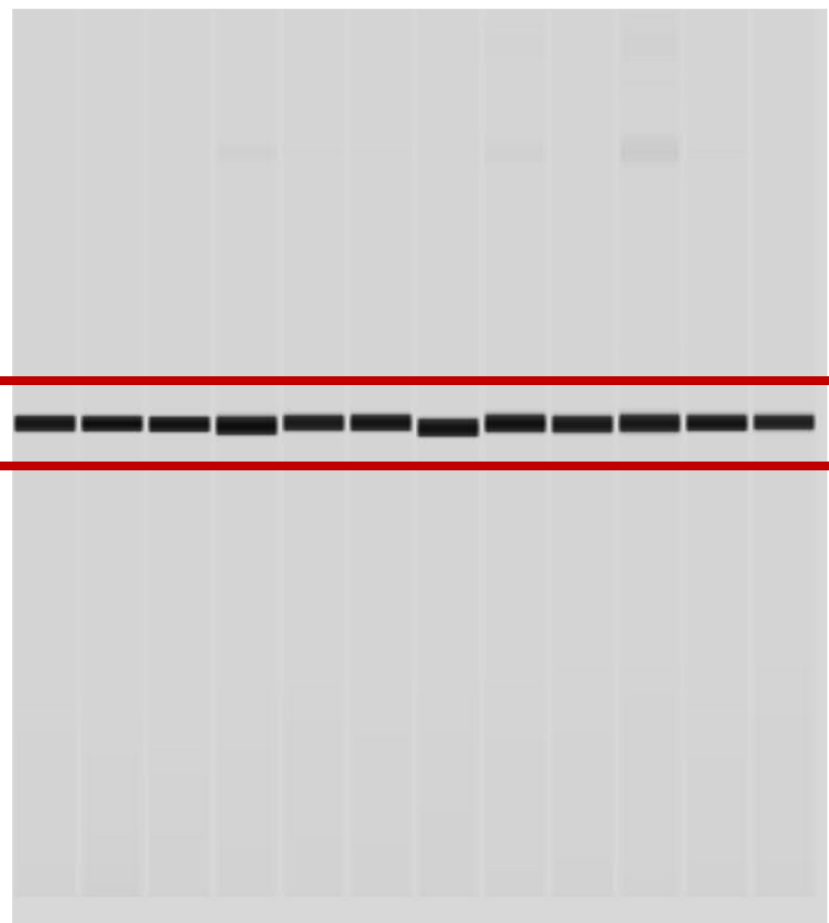

(S6 Fig)

Supplement: S6 Fig — Abbreviations: CTL, control; CLO, clopidogrel; TIC, ticagrelor; (A) S5D Fig. (B) Full-length α-EGR1 blots, from which a cropped portion (red rectangle) was taken to generate the EGR1 portion of S5D Fig. (C) Full-length α-β-Actin blots, from which a cropped portion (red rectangle) was taken to generate the β-Actin portion of S5D Fig. (PDF) [file pone.0218934.s006.pdf]
